# Molecular basis of ClC-6 function and its impairment in human disease

**DOI:** 10.1126/sciadv.adg4479

**Published:** 2023-10-13

**Authors:** Bing Zhang, Sensen Zhang, Maya M. Polovitskaya, Jingbo Yi, Binglu Ye, Ruochong Li, Xueying Huang, Jian Yin, Sebastian Neuens, Tom Balfroid, Julie Soblet, Daphné Vens, Alec Aeby, Xiaoling Li, Jinjin Cai, Yingcai Song, Yuanxi Li, Marco Tartaglia, Yang Li, Thomas J. Jentsch, Maojun Yang, Zhiqiang Liu

**Affiliations:** ^1^ Shanghai Key Laboratory of Maternal Fetal Medicine, Shanghai Institute of Maternal-Fetal Medicine and Gynecologic Oncology, Department of Anesthesiology, Clinical and Translational Research Center, Shanghai First Maternity and Infant Hospital, School of Medicine, Tongji University, 201204 Shanghai, China.; ^2^ Ministry of Education Key Laboratory of Protein Science, Beijing Advanced Innovation Center for Structural Biology, Beijing Frontier Research Center for Biological Structure, Tsinghua-Peking Center for Life Sciences, School of Life Sciences, Tsinghua University, 100084 Beijing, China.; ^3^ Leibniz-Forschungsinstitut für Molekulare Pharmakologie (FMP), 13125 Berlin, Germany.; ^4^ Max-Delbrück-Centrum für Molekulare Medizin (MDC), 13125 Berlin, Germany.; ^5^ Department of Genetics, Hôpital Universitaire des Enfants Reine Fabiola, Université Libre de Bruxelles (ULB), Brussels, Belgium.; ^6^ Department of Pediatric Neurology, Hôpital Universitaire des Enfants Reine Fabiola, Université Libre de Bruxelles (ULB), Brussels, Belgium.; ^7^ Department of Genetics, Hôpital Erasme, Université Libre de Bruxelles (ULB), Brussels, Belgium.; ^8^ Interuniversity Institute of Bioinformatics in Brussels, Université Libre de Bruxelles (ULB), Brussels, Belgium.; ^9^ Pediatric Intensive Care Unit, Hôpital Universitaire des Enfants Reine Fabiola, Université Libre de Bruxelles (ULB), Brussels, Belgium.; ^10^ Wuya College of Innovation, Shenyang Pharmaceutical University, 110016 Shenyang, China.; ^11^ Key Laboratory of Receptor Research, Shanghai Institute of Materia Medica, Chinese Academy of Sciences, 201203 Shanghai, China.; ^12^ Institute for Cognitive Neurodynamics, School of Mathematics, East China University of Science and Technology, 200237 Shanghai, China.; ^13^ Molecular Genetics and Functional Genomics, Ospedale Pediatrico Bambino Gesù, IRCCS, 00146 Rome, Italy.; ^14^ National Clinical Research Center for Aging and Medicine, Huashan Hospital, Fudan University, Shanghai 200040, China.; ^15^ NeuroCure Cluster of Excellence, Charité Universitätsmedizin Berlin, 10117 Berlin, Germany.; ^16^ Cryo-EM Facility Center, Southern University of Science & Technology, 518055 Shenzhen, Guangdong, China.

## Abstract

ClC-6 is a late endosomal voltage-gated chloride-proton exchanger that is predominantly expressed in the nervous system. Mutated forms of ClC-6 are associated with severe neurological disease. However, the mechanistic role of ClC-6 in normal and pathological states remains largely unknown. Here, we present cryo-EM structures of ClC-6 that guided subsequent functional studies. Previously unrecognized ATP binding to cytosolic ClC-6 domains enhanced ion transport activity. Guided by a disease-causing mutation (p.Y553C), we identified an interaction network formed by Y553/F317/T520 as potential hotspot for disease-causing mutations. This was validated by the identification of a patient with a de novo pathogenic variant p.T520A. Extending these findings, we found contacts between intramembrane helices and connecting loops that modulate the voltage dependence of ClC-6 gating and constitute additional candidate regions for disease-associated gain-of-function mutations. Besides providing insights into the structure, function, and regulation of ClC-6, our work correctly predicts hotspots for *CLCN6* mutations in neurodegenerative disorders.

## Teaser

Structure-based studies of the ClC-6 Cl^-^/H^+^ exchanger provide insight into CLC gating and its alteration in neurological disease.

## INTRODUCTION

CLC Cl^−^ channels and Cl^−^/H^+^ exchangers are membrane proteins that are present in almost all prokaryotic and eukaryotic cells. CLCs are essential for a wide range of physiological processes, such as the maintenance of membrane potential, transepithelial transport, and control of ion homeostasis along the endocytic pathway (*1*–*3*). Mutations in *CLCN* genes are associated with various human diseases, including myotonia, Bartter syndrome, Dent’s disease, osteopetrosis, retinal degeneration, early-onset neurodegenerative disorders, lysosomal storage disease, and hyperaldosteronism (*1*, *4*). In mammals, the CLC family comprises nine members sharing the same basic protein structure (*1*, *5*). CLCs can be divided into two distinct functional groups: Cl^−^ channels (ClC-1, ClC-2, ClC-Ka, and ClC-Kb) that mediate passive Cl^−^ flow, and chloride-proton exchangers (ClC-3 to ClC-7) that display a 2Cl^−^/1H^+^ stoichiometry (*2*).

ClC-6 is predominantly expressed in the late endosomal compartments in the nervous system (*6*). Disruption of ClC-6 in mice causes moderate neuronal lysosomal storage, which occurs mainly in axon initial segments (*6*). ClC-6 KO (*Clcn6*^−/−^) mice display reduced pain sensitivity and mild nonspecific cognitive abnormalities resembling mild forms of human neuronal ceroid lipofuscinosis (NCL) (*6*). The phenotype of *Clcn6*^−/−^ mice is much milder than those of *Clcn3*^−/−^ (*7*) and *Clcn7*^−/−^ (*8*) mice, which display severe neurodegeneration. In humans, the pathogenetic role of ClC-6 dysregulation has been firmly established by a recent report of a gain-of-function (GoF) mutation (p.Y553C) in a severe early-onset neurodegenerative disorder (*4*). The mutant exchanger displays markedly larger currents than wild-type (WT) ClC-6 and, when overexpressed in transfected cells, elicits the formation of giant Lysosome-associated membrane protein 1 (LAMP1)-positive vacuoles. Generation of these vesicles is dependent on the Cl^−^/H^+^ exchange activity of ClC-6. Hence, both loss-of-function (LoF) and GoF mutations can cause neuronal pathology. They reveal a critical role of ClC-6 in endolysosomal function (*4*, *6*).

The ClC-6 cDNA was first cloned in the 1990s (*9*). However, because the encoded protein resides in endosomes (*6*), early attempts to record ClC-6 currents were frustrated (*9*, *10*). Later, benefitting from partial plasma membrane expression of N-terminally tagged green fluorescent protein (GFP)–ClC-6, small outwardly rectifying currents of ClC-6 were recorded and confirmed by mutagenesis (*11*). However, they lacked the time-dependent gating process of other CLC exchangers (*12*–*15*) that is most obvious with the very slow opening of ClC-7 (*12*). Only recently, slow gating of WT ClC-6 was observed at rather extreme cytoplasmic positive voltages (*16*). Reassuringly, the addition of GFP to the ClC-6 N terminus did not affect current properties (*16*).

Here, we investigated the structural basis of ClC-6 function and mechanisms of pathogenic mutations. We resolved cryo–electron microscopy (cryo-EM) structures of ClC-6, adenosine 5′-triphosphate (ATP)–bound ClC-6, and the disease-causing mutant Y553C (*4*), and explored functional characteristics of ClC-6, including a previously unrecognized activation by cytosolic ATP. Together with the identification and characterization of a de novo *CLCN6* pathogenic variant (c.1558A>G, p.T520A), our results firmly establish a connection between a structural network by Y553/F317/T520 and the pathogenesis of early-onset neurodegeneration. The Y553/F317/T520 interaction network involved in ClC-6 gating guided us to further identify essential roles of interactions between intramembrane helices and their connecting loops, including P/Q linker and I/J linker, for the gating of ClC-6. These findings greatly enhance our understanding of the structure and function of ClC-6 and of its aberrant activation in human disease.

## RESULTS

### Structure of human ClC-6

**Fig. 1.**
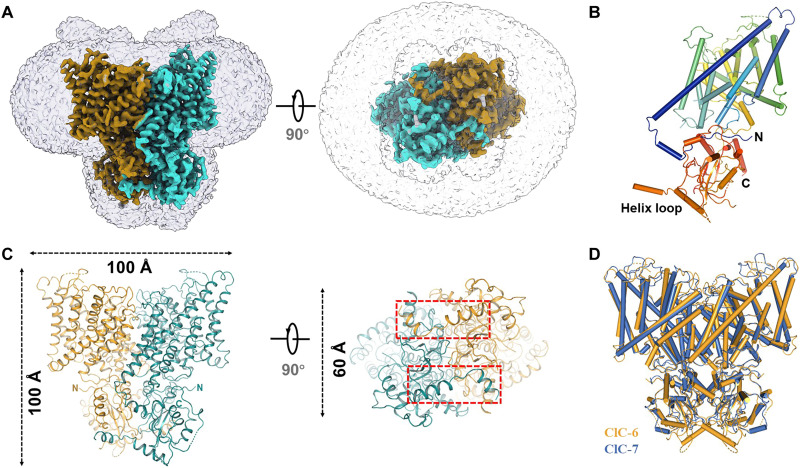
Cryo-EM structure of human ClC-6. (**A**) Cryo-EM map for the ClC-6 viewed from side (left) and top (right). The two different subunits of the dimer were highlighted by different colors. (**B**) Cartoon representation of the ClC-6 monomer, colored by structural element. (**C**) Overall structure of ClC-6 viewed from side (left) and top (right), color codes for subunits were the same as those in (A), and the long disordered region (649 to 677) is circled with red dashed boxes. (**D**) Structure superimposition of ClC-6 with ClC-7. Color codes ClC-6 and ClC-7 were indicated.

To better understand the molecular basis of ClC-6 function, we performed structural studies by cryo-EM. We purified human ClC-6 proteins from overexpressing mammalian cells through Strep-tag II affinity chromatography and reconstituted them in digitonin micelle buffer for further cryo-EM study. The structure of ClC-6 was determined at 3.5-Å resolution (Fig. 1 and fig. S1).

Akin to other CLC structures (*17*–*19*), ClC-6 adopts a dimeric architecture with each monomer containing a transmembrane domain (TMD) composed of 18 intramembrane helices and a cytoplasmic part composed of an N-terminal domain and two C-terminal cystathionine β-synthase (CBS) domains. The TMD region of the dimer consists of two independent hourglass-shaped transport pathways with a narrow selectivity filter. The beginning of the first transmembrane helix (helix B) displays a positively charged stretch (KKGRR) (fig. S2) that has been reported to be important for the association of ClC-6 with detergent-resistant membranes and its trafficking and segregation from ClC-7 in the endolysosomal pathway (*20*). Compatible with such a role, this nonconserved motif adopts a lipid-facing conformation (figs. S2 and S3), suggesting a strong potential to form interactions with the hydrophilic head tail of lipids. Intriguingly, a long disordered region (649 to 677) between the two CBS domains, which is unique for ClC-6 (fig. S2), spans from the bottom of one monomer to the neighboring monomer (Fig. 1C).

### Ion translocation pathway of ClC-6

The ion transport pathway of ClC-6 resembles an hourglass and is constricted by the selectivity filter in the middle of the TMD region. The N termini of α helices F, N, and D point toward the transport pathway and generate an electrostatically positive environment to attract anions. As in other CLC proteins (*21*), two distinct chloride binding sites (external site, S_ext_; central site, S_cen_) are distinguished (fig. S4A). S_ext_ is formed by the main-chain amide of F489 and V490 from helix N, and K199 and E200 from helix F, while the S_cen_ is surrounded by side-chain hydroxyl groups of Y576 and S157 and the main-chain amide group of L488 and F489 (fig. S4B). We did not observe electron density at an internal site (S_int_) that has been observed in *Escherichia coli* (*Ec*ClC-1) (*21*) and other CLCs. The side chain of the “gating glutamate” of ClC-6 (E200, *E*_gate_) is oriented toward the extracellular side, reminiscent of the position of *E*_gate_ of ClC-7 in the ClC-7/Ostm1 structure (*17*, *18*) or of the *Ec*ClC-1*^E148Q^* mutant (*21*), in which the glutamine is thought to mimic a protonated glutamate (fig. S4C) (*18*).

### Electrophysiological characteristics of ClC-6

**Fig. 2.**
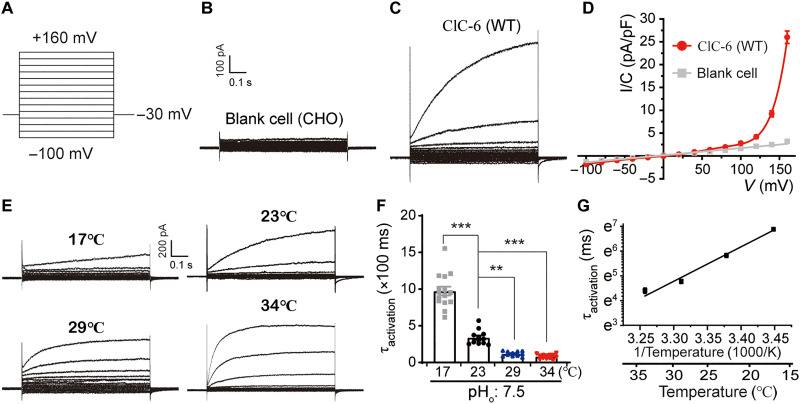
Electrophysiological characteristics of ClC-6. (**A** to **C**) Whole-cell recording protocol (A) and representative current traces of untransfected Chinese hamster ovary (CHO) cells (B, *n* = 9/4, indicates independent cells/batches, the same hereinafter) and CHO cells overexpressing WT ClC-6 (C, *n* = 24/7), with bath solution containing 161 mM Cl^−^ (pH_o_ 7.5). After holding at −30 mV, cells were clamped between −100 to +160 mV in 0.8-s steps of 20 mV (A). (**D**) Current density [current magnitude/cell capacitance (*I/C*)]-voltage (*V*) relationship (abbreviated as *I/C-V*) corresponding to results are shown in (B) and (C). (**E** to **G**) Influence of temperature on activation kinetics of ClC-6. Representative current traces at different temperatures (E; 17°, 23°, 29°, 34°C, the corresponding *n* = 16/4, 12/3, 11/3, and 13/3). The activation time constant (τ_activation_) at +160 mV of ClC-6 under different temperature was shown as bar graphs (F) and fitted by Arrhenius equation (G). Data were represented as means ± SEM, and one-way ANOVA with post hoc Bonferroni tests (F) was performed. **P < 0.01 and ***P < 0.001.

To study the transport properties of ClC-6, we used GFP–ClC-6 fusion constructs that are partially mislocalized to the plasma membrane (*11*). Whereas earlier studies have reported almost instantaneous ClC-6 currents when clamping membranes up to +100 mV, more recent use of rather extreme voltage steps up to +180 mV revealed a slow gating process (*16*) that had previously been observed with the Y553C mutant under less positive voltages (*4*). Also, in our hands, slowly activating currents were obtained when stepping to voltages > +120 mV (Fig. 2, A to D). As expected, these currents strongly depended on extracellular [Cl^−^]_o_ (fig. S5, A to C).

The slow gating of CLC channels (*22*, *23*) and exchangers (*12*) shows a strong temperature dependence. Likewise, increasing the temperature (from 17° to 34°C) markedly shortened the time course of ClC-6 activation (Fig. 2, E and F). The time constant at +160 mV at different temperatures was well fitted by the Arrhenius equation (Fig. 2G), yielding a mean activation enthalpy (∆*H*^‡^) of approximately 110 kJ mol^−1^, corresponding to a temperature coefficient (*Q*_10_) of approximately 4.5.

CLC transporters exchange 2Cl^−^ for 1H^+^ ion, which likely involves protonation and movement of the side chain of *E*_gate_ (E200 in ClC-6) (*24*–*28*). *E*_gate_ is located close to the center of the pore and is thought to block the transport pathway unless it is protonated (*19*, *28*). H^+^ ions not only influence Cl^−^/H^+^ exchange rates (reflected in pH-dependent amplitudes of electrical currents) by serving as transported substrates but may also modulate ion transport by binding to other residues. Whereas previous studies demonstrated that CLC antiporter currents are decreased by extracellular (luminal) acidification (*1*, *11*, *12*, *14*, *15*, *29*, *30*), we found, in agreement with a recent report (*16*), that ClC-6 currents were substantially increased with acidification and decreased with alkalinization when measured at potentials >+120 mV (fig. S5, D to J).

### Intersubunit and interdomain interactions play critical roles in ClC-6 function

**Fig. 3.**
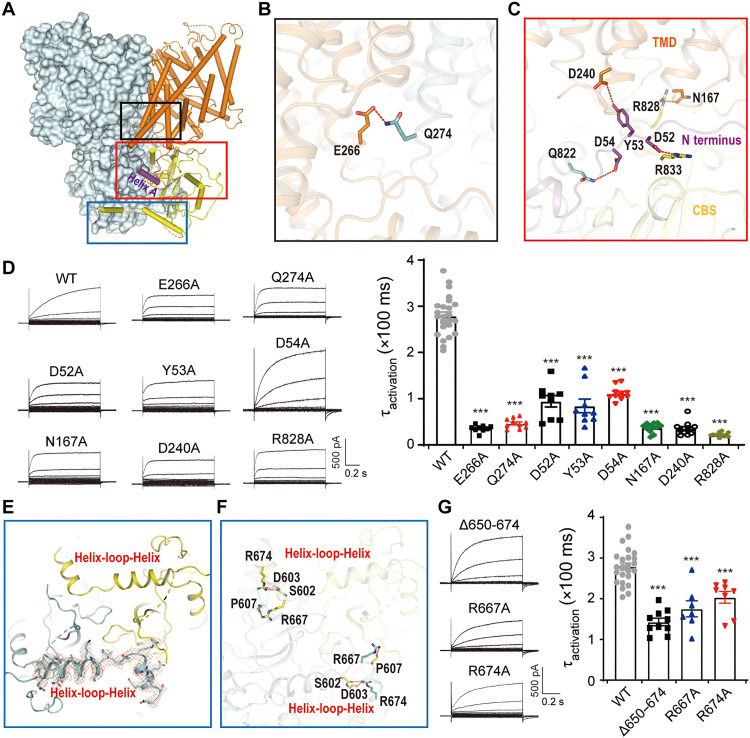
Roles of intersubunit and interdomain interactions for ClC-6 function. (**A**) Overall structure of ClC-6 with one monomer shown in the surface style. The interaction zones for functional analysis were circled with colored boxes (black: dimeric interactions within TMD; red: N-terminal–mediated polar interactions; blue: C-terminal protruding into opposite CBS domain). (**B** and **C**) Magnified view for dimeric interactions within TMD (B) and N-terminal–mediated polar interactions (C). The interaction was shown as dashed line. (**D**) Representative current traces and τ_activation_ of WT ClC-6 and mutants including E266A (*n* = 10/3), Q274A (*n* = 9/3), D52A (*n* = 9/3), Y53A (*n* = 9/3), D54A (*n* = 10/3), N167A (*n* = 12/3), D240A (*n* = 12/3), and R828A (*n* = 12/3). (**E**) A helix-turn-helix sequence between the CBS domains of one subunit protrudes into the opposite CBS. The cryo-EM density for C terminus was shown as red mesh contoured at 8σ threshold. (**F**) Magnified view for the C-terminal–mediated interactions. The interactions were shown as dashed lines. (**G**) Representative current traces and τ_activation_ of ClC-6 mutants including Δ650–674 (*n* = 10/3), R667A (*n* = 7/2), and R674A (*n* = 8/2). Data were represented as means ± SEM, and one-way ANOVA with post hoc Bonferroni tests (D and G) was performed. ***P < 0.001.

The dimeric ClC-6 structure occupies a volume of 110 Å × 100 Å × 70 Å (Fig 1C). At the dimer interface, E266 in helix H from one monomer forms hydrogen bonds with Q274 at the beginning of helix I from the opposite monomer (Fig. 3, A and B). Similar to findings with ClC-7/Ostm1 (*17*, *18*), part of the N terminus of ClC-6 (residues 46 to 70, preceding helix A) protrudes into the cleft between the TMD and CBS domains of the same monomer (Fig. 3, A and C). It forms substantial polar interactions with both structures. Residues D52, Y53, and D54 from the N-terminal region interact with R833 and Q822 of the CBS domain as well as D240 from the TMD, while N167 forms hydrogen bonds with R828 from the CBS domain (Fig. 3C). We explored whether these interactions have a critical role in the gating of ClC-6 by mutating representative-interacting residues at the dimer interface (TMD: E266 and Q274) and the TMD/N terminus/CBS interaction zone (D52, D54, D240, N167, and R828) to alanine. All these mutations accelerated voltage-dependent activation of currents (Fig. 3D).

At the bottom of the CBS domain, a helix-turn-helix region from one monomer protrudes to the neighboring monomer and forms interactions (R667–P607, R674–S602) that stabilize the CBS domain (Fig. 3, A, E, and F, and fig. S6). Among CLC exchangers, only ClC-6 displays such a large insertion of additional amino acids between CBS1 and CBS2 (fig. S2). Partial deletion of this stretch (Δ650–676) and mutations of key interactors (R667A and R674A) accelerated the activation kinetics of ClC-6 (Fig. 3G), implying an important role of CBS domain interactions in ClC-6 gating.

### Effect of ATP binding on ClC-6 function

**Fig. 4.**
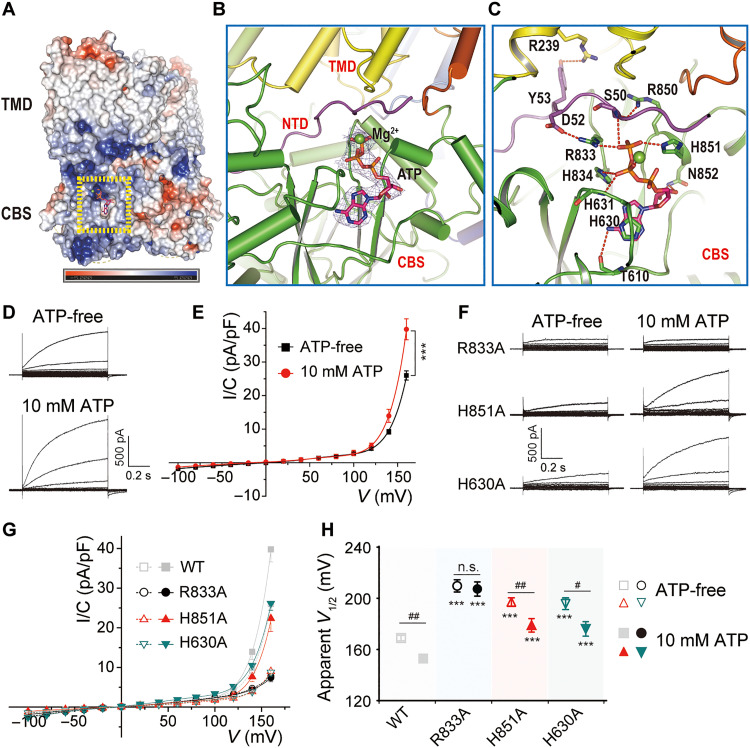
Structure and effect of ATP binding on ClC-6. (**A**) Structure of ClC-6 in complex with ATP. ATP-binding site highlighted by yellow dashed box. (**B** and **C**) Magnified view of ATP-binding site in ClC-6. The cryo-EM density for ATP molecule shown as blue mesh contoured at 8σ threshold (B). Key residues interacting with ATP shown as sticks (C). (**D** and **E**) Representative current traces and *I/C*-*V* curves of ClC-6 without or with 10 mM ATP in the pipette solution (*n* = 9/3). (**F** and **G**) Representative current traces and *I/C*-*V* curves of mutants predicted to neutralize ATP binding without [empty icons in (G); *n* = 11/3, 11/3, and 10/3 for R833A, H851A, and H630A] or with 10 mM ATP [filled icons in (G); *n* = 12/3, 9/3, and 8/2 for R833A, H851A, and H630A) in the pipette solution. (**H**) Influence of ATP and mutations on the voltage dependence of ClC-6 that was quantified by determining an apparent *V*_1/2_. Data were represented as means ± SEM, and unpaired Student’s *t* tests (E) and two-way ANOVA with post hoc Bonferroni tests (H) were performed. ***P < 0.001 [in (H), compared to WT ClC-6 with an equivalent experimental condition) and #*P *< 0.05, ##*P *< 0.01, ###*P* < 0.001. n.s., not significant.

ATP binds to CBS domains of several CLC transporters and channels and changes voltage-dependent gating of ClC-1 and ClC-2 Cl^−^ channels (*31*, *32*) and of ClC-3−5 2Cl^−^/H^+^ exchangers (*33*), but no corresponding data are available for ClC-6. When we purified ClC-6 in the presence of 2 mM ATP, the resulting structure revealed (at 3.4-Å resolution) that ATP was bound to its CBS domains (Fig. 4, A to C, and fig. S7). The adenine base of ATP interacts through π-stacks with the side chain of H630 on each CBS1 (Fig. 4C). The triphosphate group is coordinated by residues from both CBS domains of the same subunit and its N-terminal domain. Interacting structures include the side chains of R833, H851, and H834, and the main chain of S50 (Fig. 4C).

The amplitude of ClC-6 currents was substantially increased with 10 mM compared to no ATP in the pipette solution (Fig. 4, D and E). Mutants predicted to disrupt ATP binding (R833A, H851A, and H630A) markedly decreased amplitudes of ClC-6 currents also in the nominal absence of ATP (Fig. 4, F to H, and fig. S8, A and B). This suggests that these mutations either have functional effects independent of ATP binding or that residual cytosolic ATP remaining in whole-cell recordings with ATP-free pipette solution is high enough to bind WT, but not mutated binding pockets. Compatible with the second hypothesis, ATP binding appeared completely abolished in the R833A mutant, which did not respond to 10 mM ATP, but was only reduced with H851A and H630A, which still responded to ATP (Fig. 4, F to H, and fig. S8, A and B).

### Disease-causing mutations reveal structural interactions in ClC-6 gating

Recently, heterozygous p.Y553C *CLCN6* mutations were found in three independent individuals presenting with severe early-onset neurodegeneration (*4*). By shifting the voltage dependence of ClC-6 to more negative potentials (*16*), as confirmed here (fig. S9, A to C), the mutation notably increases currents at less positive, more physiological voltages, providing a GoF behavior. Overexpression of the mutant in mammalian cells generates giant LAMP1-positive vesicles (*4*).

**Fig. 5.**
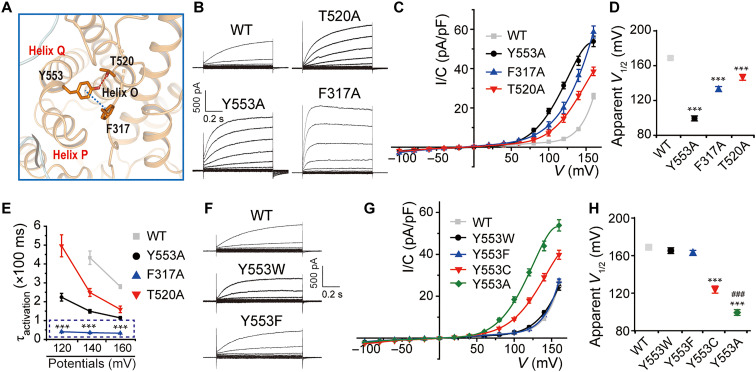
Role of Y553/F317/T520 interaction network for the voltage dependence of ClC-6. (**A**) Magnified view for the interactions among Y553, F317, and T520. The interactions shown as dashed lines. (**B** and **C**) Representative current traces and *I/C-V* curves of WT ClC-6 and mutants including Y553A (*n* = 11/3), F317A (*n* = 12/3). and T520A (*n* = 7/3). (**D**) Apparent *V*_1/2_ was compared between groups shown in (B) and (C). (**E**) Value of τ_activation_ at 120, 140, and 160 mV was compared between groups shown in (B) and (C). (**F**) Representative current traces of WT, Y553W (*n* = 6/2), and Y553F (*n* = 11/3). (**G**) *I/C*-*V* curves of WT ClC-6 and mutants including Y553W, Y553F, Y553C, and Y553A. (**H**) Apparent *V*_1/2_ was compared between groups shown in (G). Data were represented as means ± SEM, and one-way ANOVA with post hoc Bonferroni tests (D, E, and H) were performed. ***P < 0.001 (versus WT ClC-6) and ^###^*P* < 0.001 (versus Y553C mutant).

To gain structural insights into the pathogenic mechanism, we solved the cryo-EM structure of Y553C mutant at 3.4-Å resolution. Except for the single-residue alteration of Y553, the structures of WT and Y553C appeared identical. No alterations were seen in the ion conduction pathway and at the dimer interface (fig. S9, D and E). This may be expected since both WT and mutant, voltage-gated transporters do not transport ions at 0 mV, that is at the conditions used for obtaining cryo-EM structures. However, we observed that helix Q residue Y553 is in close proximity to F317 and T520 (Fig. 5A) (located in the I/J linker and helix O, respectively). Mutating any of these amino acids to alanine notably shifted the voltage dependence to more negative voltages (Fig. 5, B to D). F317A also markedly accelerated voltage-induced activation (Fig. 5E). By contrast, mutants retaining the aromatic ring of the side chain of Y553 and F317, including Y553F, Y553W, F317Y, and F317W, had no significant impact on the voltage dependence (Fig. 5, F to H, and fig. S10, A to C), indicating a contribution of π-π–conjugated bond between Y553 and F317 to the observed functional changes. Y553A had a significantly larger effect on the voltage dependence than Y553C (Fig. 5, G and H), suggesting an effect of steric hindrance by the side chain of Y553 on gating-associated structural rearrangements. F317Y and F317W also diminish the activation time constant similar to that of F317A mutant, which indicates that voltage dependence and kinetics of gating are differentially regulated by these interactions (fig. S10D). Mutations that weaken interactions in the Y553/F317/T520 network, including the disease-causing mutant Y553C, induce large shifts in the voltage dependence that increase currents at presumably more physiological voltages.

In the course of this work, we got access to a patient with a severe neurological disorder. She was found to be heterozygous for a previously unreported de novo *CLCN6* missense change (c.1558A>G, p.T520A, NM_001286.3), which was predicted as damaging by several in silico tools and classified as pathogenic based on the American College of Medical Genetics and Genomics (ACMG) criteria (*34*) (supplementary case report). Strikingly, this is exactly the same mutation we had already investigated functionally based on the structural prediction that T520 is close to the disease-associated Y553 residue. Beginning at 16 months of age, the 6-year-old girl presented recurrent paroxysmal episodes characterized by hypopnea/apnea, requiring several periods of invasive ventilation, loss of consciousness, and sometimes tonic or myoclonic movements of the upper limbs (for clinical details, see supplementary case report and table S1). Interictal electroencephalogram showed slow wave activity compatible with mild to moderate encephalopathy but no epileptic activity. She displayed global developmental delay and general muscular hypotonia, which resulted in an inability to walk without support. Magnetic resonance imaging revealed white matter hypomyelination and spinal cord swelling among other abnormalities (fig. S11A). These symptoms broadly overlapped with those observed in the three unrelated individuals heterozygous for the pathogenic *CLCN6* missense substitution (p.Y553C) (*4*). Akin to Y553C, the T520A mutant not only shifted voltage-dependent gating (Fig. 5, B and D) but also generated giant LAMP1-positive, lysosome-like vacuoles when overexpressed in transfected cells (fig. S11B). When ClC-6 was converted from a 2Cl^−^/H^+^ exchanger into a pure Cl^−^ conductance by the uncoupling E200A mutation, which by itself leads to moderately enlarged vesicles (*4*) and is associated with a distinct neurological disorder (*35*, *36*), additional insertion of the T520A mutation (E200A/T520A) no longer enlarged vacuoles to the same degree (fig. S11B). Meanwhile, large vacuoles were not generated when T520A was inserted into a transport-deficient “proton glutamate” mutation E267A (E267A/T520A) (fig. S11B). These “td” mutants strongly reduce (*11*, *12*, *37*, *38*) but do not completely abolish (*16*, *39*) ion transport of mammalian CLC exchangers. It remains unclear whether they also abolish proton coupling of Cfl transport as has been observed with *Ec*ClC-1 (*40*). Thus, the enlargement of vacuoles likely depends to a large degree on pH gradient-driven Cl^−^ uptake into their lumen, followed by osmotic swelling and inhibition of vesicle budding and scission (*4*). Hence, our high-resolution structure identified a functionally important residue that was later found in human genetic disease.

### Role of P/Q linker in gating

**Fig. 6.**
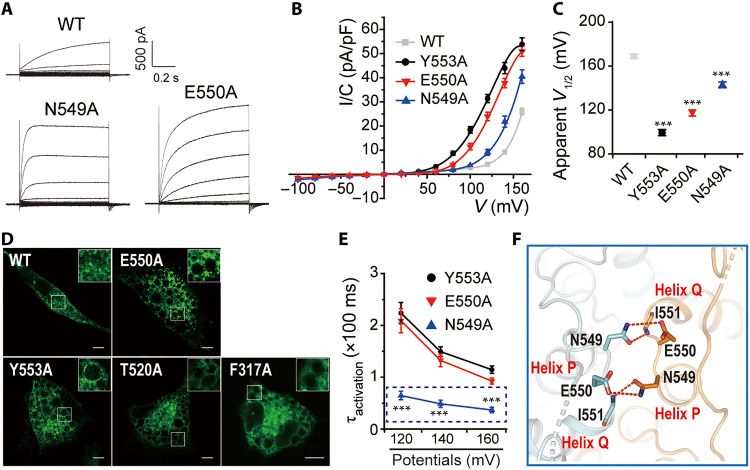
Role of P/Q linker in gating. (**A** and **B**) Representative current traces and* I/C-V* curves of WT ClC-6, N549A (*n* = 11/3), and E550A (*n* = 11/3) mutants. (**C**) Apparent *V*_1/2_ were compared among groups shown in (B). (**D**) Images displaying GFP fluorescence of CHO cells overexpressing N-terminally GFP-tagged WT ClC-6 and mutants including E550A, Y553A, T520A, and F317A, respectively. Scale bars, 10 μm. (**E**) Value of τ_activation_ at 120, 140, and 160 mV was compared among Y553A, E550A, and N549A. (**F**) Magnified view of interactions between N549 and E550. The interactions were shown as dashed lines. Data were represented as means ± SEM, and one-way ANOVA with post hoc Bonferroni tests (C and E) was performed. ***P < 0.001.

Y553 is close to the N-terminal end of helix Q. Motion of the P/Q linker contributes to substrate-driven conformational changes of the *E. coli* exchanger *Ec*ClC-1 (*41*). Restricting the flexibility of the P/Q linker reduces the transport activity of *Ec*ClC-1 (*42*). Our alanine scanning mutagenesis of the P/Q linker (residues 546 to 553) identified two mutants, N549A and E550A, which shifted the voltage dependence to negative potentials and substantially increased current amplitudes (Fig. 6, A to C). Both the shifted voltage dependence of E550A and the generation of giant vacuoles were reminiscent of disease-causing Y553A and T520A mutants and of the F317A mutant (Fig. 5, B to D, and 6, A to D). In addition, N549A accelerated the activation kinetics of ClC-6 (Fig. 6E). N549 from one monomer interacts with the backbone nitrogen of E550 from another monomer, indicating that these interactions have an important role in stabilizing the architecture of P/Q linker (Fig. 6F). Collectively, these results indicate an essential role of residues at or near the P/Q linker, such as Y553, E550, and N549, in modulating the voltage dependence of ClC-6.

### Interactions of the I/J linker with TMD helices modulate ClC-6 activation kinetics

**Fig. 7.**
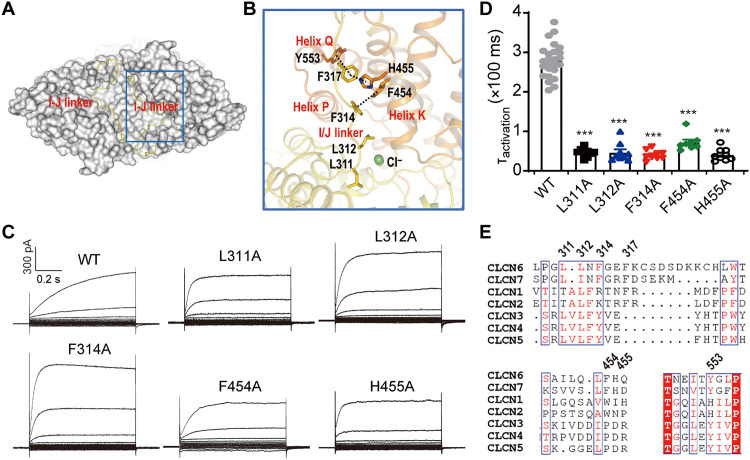
Interactions of the I/J linker with TMD helices modulate ClC-6 activation kinetics. (**A**) Surface representation of ClC-6 structure to clarify the position of I/J linker (Yellow ribbon). (**B**) Magnified view for the interactions between I/J linker and the surrounding residues, the interactions were shown as dashed lines. (**C** and **D**) Representative current traces and τ_activation_ of WT ClC-6 and mutants including L311A (*n* = 9/3), L312A (*n* = 9/3), F314A (*n* = 9/3), F454A (*n* = 8/2) and H455A (*n* = 9/3). (**E**) Sequence alignment of the I/J linker and the surrounding residues of ClC-6 with other CLCs. Data represented as means ± SEM; one-way ANOVA with post hoc Bonferroni tests (D) was performed. ***P < 0.001.

F317, which shifted the voltage dependence of ClC-6 and notably accelerated activation (Fig. 5), is located in the I/J linker (residues 300 to 330), which comprises a long loop situated above the ion translocation pathway. It displays several interactions with surrounding residues (Fig. 7, A and B). For instance, F317 interacts with H455 from helix K and Y553 from helix Q at the same time, whereas residue F314 interacts with the neighboring F454 from helix K. L311 and L312 reside above the chloride coordination sites and form hydrophobic interactions with nearby helices, such as helices B, E, and K (Fig. 7B). To further probe potential roles of interactions between the I/J linker and intramembrane helices, we analyzed several mutants that disrupt these interactions. Mutants such as L311A, L312A, F314A, F454A, and H455A consistently accelerated voltage-dependent activation (Fig. 7, C and D). Residues L311 and L312 are conserved among mammalian CLC exchangers, whereas F314, F317, F454, and H455 are conserved only between ClC-6 and ClC-7 (Fig. 7E). This suggested that similar I/J linker-mediated interactions may occur in other CLC antiporters. Together, these results suggested that the I/J linker coordinates with TMD helices to modulate the activation kinetics of ClC-6.

### Interactions between transmembrane helices affect ClC-6 gating

**Fig. 8.**
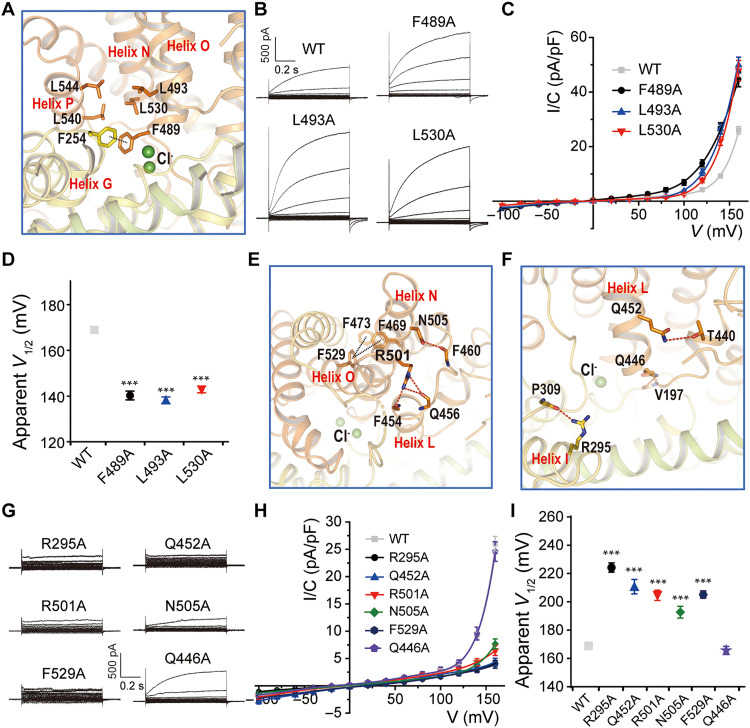
Role of interhelical interactions in ClC-6 gating. (**A**) Magnified view for the inter-helical connections between helix O and G, N, and P. Residues that contribute to interhelical contacts were shown as sticks. (**B** and **C**) Representative current traces and *I/C*-*V* curves of WT ClC-6 and mutants including F489A (*n* = 9/3), L493A (*n* = 10/3), and L530A (*n* = 9/3). (**D**) Apparent *V*_1/2_ were compared between groups shown in (B) and (C). (**E** and **F**) Magnified view for the interhelical connections that are far from the Cl^−^ binding sites. Residues that contribute to interhelical contacts were shown as sticks. (**G** and **H**) Representative current traces and *I/C*-*V* curves of mutants including R295A (*n* = 7/2), Q452A (*n* = 8/3), R501A (*n* = 8/3), N505A (*n* = 8/3), F529A (*n* = 9/3), and Q446A (*n* = 5/2). (**I**) Apparent *V*_1/2_ was compared between groups shown in (G) and (H). Data were represented as means ± SEM, and one-way ANOVA with post hoc Bonferroni tests (D and I) was performed. ***P < 0.001.

No obvious voltage sensors are observed in CLC proteins. The voltage-dependence CLC ion transport is conferred by several determinants, including permeant anions, protons, and the movement of the charged side chain of the *E*_gate_ with which these ions interact (*1*). Many mutations in TMDs of CLC Cl^−^ channels affect voltage-dependent gating (*43*–*45*), suggesting that it involves conformational changes larger than an isolated movement of the side chain of *E*_gate_. Even individual exchange cycles of the bacterial 2Cl^−^/H^+^-exchanger *Ec*ClC-1 may require movements of intramembrane helices (*41*, *42*, *46*). On the basis of structural predictions, we mutated candidate residues within the TM region of ClC-6, focusing at first on regions close to the permeation pathway. Residue F489 resides in the N terminus of helix N close to the S_ext_ and S_cen_ chloride binding sites (Fig. 8A); F489 also forms π-π interactions with the neighboring F254 from helix G (Fig. 8A). Converting F489 to alanine (F489A) increased current amplitudes by shifting the voltage dependence to the left (Fig. 8, B to D). A hydrophobic interaction cluster was observed between L493 from helix N, L530 from helix O, L540 and L544 from helix P (Fig. 8A). L493A and L530A left-shifted the voltage dependence of ClC-6 similar to F489A (Fig. 8, B to D).

We next examined residues participating in interhelical interactions that are far from the Cl^−^ binding sites. Specifically, residues F529 from helix O, R501 and N505 from the distal end of helix N, Q452 and Q446 from helix L, and R295 from helix I were separately mutated to alanine (Fig. 8, E and F). Five mutants, R295A, Q452A, R501A, N505A, and F529A, strongly decreased current amplitudes, whereas Q446A had no effect (Fig. 8, G to I). The apparent decrease in currents of the five mutants might be attributed to a shift of the voltage dependence to more positive voltages, as indicated by the emergence of slowly activating currents at even more extreme voltages (fig. S8, C and D). Collectively, these results suggest that changes in the relative positions of intramembrane helices, including helices I, L, N, O, and P, as well as the I/J linker, occur during ClC-6 gating.

## DISCUSSION

We report the first high-resolution structure of ClC-6, a late endosomal 2Cl^−^/H^+^ exchanger recently shown to underlie severe neurological disease when mutated (*4*, *36*). Our structural analysis correctly predicted that mutations in two residues (T520 and F317) in the vicinity of a disease-associated tyrosine (Y553) might have similar effects on voltage gating. Strikingly, we identified a previously undescribed pathogenic amino acid substitution (p.T520A) in one of these residues. It closely replicated the clinical and cell biological effects of the previously described p.Y553C mutation (*4*). These residues form a structural network involved in ClC-6 gating. We further expanded these findings to interacting residues in other loops and intramembrane helices. In addition to movements of cytosolic CBS domains, which we found to bind ATP, rearrangements of intramembrane helices and their connecting loops occur during voltage-driven gating of ClC-6.

### Gating of CLCs

Both CLC Cl^−^ channels and 2Cl^−^/H^+^-exchangers are formed by homodimers (*47*, *48*) or sometimes heterodimers (*49*–*52*) of CLC proteins. Each monomeric subunit encloses an independent ion permeation pathway. In ClC-0 from the electric ray *Torpedo* (*53*), pores are individually gated by a fast, depolarization-activated protopore gate (*54*), which is believed to be embodied by the *E*_gate_ that is located in the pore (*21*). In addition, both pores are gated together by a slow hyperpolarization-activated “common” gate (*54*). Gating depends not only on voltage but also on the concentrations of Cl^−^ and H^+^, which interact with the *E*_gate_. The voltage dependence is probably conferred by the permeant ions (*55*, *56*). Common gating of both channel- and exchanger-type CLCs remains poorly understood. It cannot be based solely on the movement of a few pore residues but requires larger conformational changes that are transmitted from one subunit to another.

With CLC exchangers, current relaxations upon voltage steps have first been observed for ClC-4 and ClC-5 (*15*). Later studies with ClC-7/Ostm1 convincingly showed slow activation by voltage (*12*). ClC-7 WT/mutant heterodimers revealed that it represents common gating (*57*), strongly suggesting that common gating also underlies the slow activation of ClC-6. This notion fits to the temperature dependence of ClC-6 that resembles that of ClC-7 [*Q*_10_ of ~4.5 for ClC-6 (Fig. 2) and *Q*_10_ ~3 for ClC-7 (*12*)]. Common conformational changes of both subunits, as necessary for common gating, likely involve a larger energy barrier between open and closed state compared to protopore gating, which mainly involves the movement of the side chain of the gating glutamate. Protopore and common gating of ClC-0 indeed display large differences in *Q*_10_ (2.2 and 40, respectively) (*23*). The intermediate value of Q_10_ for ClC-6 and ClC-7 would be compatible with either gating process. So far, however, there is no evidence for “protopore” gating of CLC exchangers. Fitting the present activation curves with two exponentials did not reveal two kinetically different gating processes. Moreover, the concept of protopore gating of CLC channels, in which the side chain of the gating glutamate swings aside upon protonation to allow the flux of thousands of ions, is difficult to extend to CLC exchangers. Here, each exchange cycle is thought to involve conformational changes of the gating glutamate (*27*, *28*). However, more recent studies suggest that even single exchange cycles might involve more widespread conformational changes (*25*, *46*).

### Role of cytoplasmic CBS domains in ClC-6 gating and regulation

Eukaryotic CLC proteins contain two CBS domains in their cytosolic tails whose functions remain incompletely understood. CBS domains of several CLC proteins bind ATP, which has diverse effects on ion transport activity (*31*–*33*, *58*–*60*). Mutations in their C termini affect gating of ClC-0 and ClC-1 (*61*, *62*). Fluorescence resonance energy transfer experiments suggest large movements of C termini of ClC-0 during common gating (*63*), but there are currently no CLC structures that support this notion. Cryo-EM studies revealed interactions of CBS domains with each other, with the TMD region, and with the N terminus. However, it remains unclear how conformational changes in CBS domains may affect gating.

In ClC-6, a segment between CBS1 and CBS2 protrudes into the C-terminal tail of the neighboring subunit. This interaction may be important for coupling both subunits during common gating. However, because no such segment is found in ClC-7, it cannot play a universal role in CLC gating. Similar to ClC-7/Ostm1 (*17*, *18*), we found that a segment of the N terminus is sandwiched between the TMD and CBS domains of the same ClC-6 subunit. Mutating several predicted interacting residues, in the TMD, N terminus, or CBS domains, again shifted the voltage dependence of gating kinetics.

ATP modulates the activity of multiple CLCs, but the precise mechanism remains unclear (*31*–*33*, *58*–*60*, *64*–*67*). The present structure (Fig. 4) reveals that also ClC-6 binds ATP and that the binding site resembles those of ClC-5 (*59*), ClC-7/Ostm1 (*18*), and maybe ClC-1 (*68*). Cytoplasmic ATP enhanced ClC-6 currents, and the R833A mutation targeting the binding pocket completely abolished this effect. Rather unexpectedly, three mutations predicted to abolish ATP binding strongly suppressed currents also in the nominal absence of ATP. While we cannot exclude that these mutations affect currents independent of ATP binding, we assume that the affinity of WT, but not mutated, binding sites is high enough to allow substantial ClC-6 activation with very low cytoplasmic ATP concentrations that may remain in the cytosol even after several minutes of dialysis with ATP-free pipette solutions. Two mutants (H851A and H630A) gave no currents in the nominal absence of ATP but could be stimulated by 10 mM ATP, suggesting that their affinity for ATP was decreased, but not abolished. A high affinity of the ClC-6 ATP-binding site is reminiscent of ClC-5 where the affinity of adenine nucleotides [K*_d_* (dissociation constant) ~ 100 μM (*59*)] is an order of magnitude lower than physiological cytosolic ATP concentrations (2 to 5 mM). Hence, constitutively bound ATP may serve a structural role (*18*, *59*). On the other hand, a physiological importance of adenine nucleotide binding is suggested by disease-related *CLCN7* mutations around the ATP-binding site (*12*, *69*) and ATP binding to endosomal ClC-3 to ClC-5 might serve to sense the metabolic state of the cell (*33*). Neither the mechanism by which ATP binding influences ClC-6 ion transport, nor its physiological role are clear at this point.

### Conformational changes in several TMD regions associated with ClC-6 gating

Our work suggests structural rearrangements in several protein regions during ClC-6 gating. Guided by a mutation (p.Y553C) associated with a severe neurodevelopmental/neurodegenerative disorder (*4*), we identified an interaction network including residues on helix Q (Y553), helix O (T520), and the I/J linker (F317). Weakening pairwise interactions by point mutations activated ClC-6 by shifting its voltage dependence to more negative voltages. We then analyzed other residues of the I/J linker and found that disrupting its interaction with helix K similarly facilitated ClC-6 opening. Last, we extended this type of analysis to residues in other ClC-6 regions. In many cases, shifts in voltage dependence and activation kinetics were observed. These results suggest that ClC-6 gating is associated with movements between TMD helices and between helices and intervening linkers, including helices I, L, N, O, and P, as well as the I/J linker.

Notably, disease-related variants in the related ClC-3 and ClC-4 exchangers occur in regions we identified here as being important for ClC-6 gating. Mutations such as ClC-3*^I607T^*, ClC-4*^I549N^* (equivalent to G*^554^* in ClC-6), ClC-4*^V550L^*, and ClC-4*^A555V^*, located in the P/Q linker and helix Q; ClC-3*^T570I^*, located in helix O, ClC-4*^P310S^*, ClC-4*^V317F^*, and ClC-4*^V317I^*, located in helix I and I/J loop cause abnormal inward currents at acidic extracellular pH and are associated with neurological disease (*70*, *71*). Hence, our conclusions may extend to other CLC antiporters. However, the question why disruptions in gating tend to cause a loss of rectification in ClC-3/-4 at acidic pH and a left shift in voltage dependency in ClC-6 and ClC-7 remains enigmatic.

### Interactions between helices and intervening loops affect gating

The present mutations, particularly those which do not change the charge, are unlikely to affect voltage sensing per se. Voltage sensing of membrane proteins relies on electrical charges in the transmembrane electrical field. In contrast to a family of cation channels (*72*), no voltage sensing–charged amino acids have been identified in CLC transmembrane regions. Voltage-dependent gating of CLCs rather depends on Cl^−^ and H^+^ ions that sense the electric field in the permeation path (*1*, *13*, *55*, *73*, *74*).

Changing interactions between different parts of the protein may change the energy barrier between the closed and the open state and/or the difference in energy between these states. Lowering the energy barrier is expected to accelerate opening, whereas lowering the energy difference may shift the voltage dependence, but not its steepness. Many mutations both accelerated gating and shifted activation threshold to more negative voltages, compatible with a stabilization of the closed state by the interactions that were disrupted. However, some mutations such as R295A and Q452A markedly shifted the voltage dependence to the right, abolishing currents at voltages that seem more physiological. Hence, both GoF and LoF amino acid substitutions, with a shift of voltage dependence toward and away from physiological voltages, respectively, were obtained.

Previous findings already suggested conformational changes of CLC proteins during common gating (*17*, *57*, *63*, *75*). These studies, however, focused on the cytosolic domains of CLCs. By interfering with interactions between amino acid pairs indicated by our structure, our work now suggests relative movements of several transmembrane helices during transporter gating. Some of the structures we predict to move during gating are believed to move also during 2Cl^−^/H^+^ exchange cycles of *Ec*ClC-1 (*42*). Notably, these two processes operate at vastly different time scales, with voltage-dependent “opening” of the transporter (*12*) enabling a very large number of exchange processes. Nonetheless, these processes are probably intimately linked. Gating of CLC channels involves *E*_gate_ (*21*, *76*), which in CLC exchangers is the site of 2Cl^−^/H^+^ exchange. Its neutralization leads to both loss of Cl^−^/H^+^ coupling and voltage dependent (*11*, *12*, *14*, *77*). Since common gating affects both subunits of the dimer, it must involve conformational changes at the interface between the subunits. These may involve the large contact surface between the TMDs, as exemplified by the E266 and Q247 pairs, and interactions between cytosolic parts of ClC-6 (Fig. 3).

### ClC-6 structure predicts mutational hotspots for neurological disease

Starting from a residue (Y553) previously found to be mutated to cysteine in three independent patients with severe childhood-onset neurodegeneration with hypotonia, respiratory insufficiency, and brain imaging abnormalities (*4*), we identified three interacting residues in its three-dimensional (3D) vicinity. Replacing these residues by alanine (F317A, T520A) affected gating similar to Y553C (Fig. 5). This suggested a loss of pairwise interactions as underlying mechanism. All three mutants accelerated ClC-6 gating and shifted the voltage dependence to less positive voltages. Unfortunately, estimates for the voltage across endolysosomal membranes vary widely. Values between −40 and +114 mV (lumen referred to cytosol, opposite to convention for plasma membrane voltages) have been reported (*78*–*81*). Voltages between ±20 mV seem reasonable (*80*). All vesicular CLCs gate open at cytosol-positive, that is lumen-negative, potentials. With the exception of ClC-6, their ion transport increases steeply at luminal voltages more negative than about −20 mV (*12*–*15*), a value agreeing with some measurements (*81*, *82*) and model calculations (*83*, *84*). However, these voltages, and particularly the lumen-positive potentials reported in some studies (*79*–*81*), are far from the threshold required for the slow voltage-dependent activation of ClC-6 (about −100 mV). The voltage dependencies of both Y553C (*4*) and the newly described T520A were shifted to the range where WT ClC-3, ClC-4, ClC-5, and ClC-7 open. This shift to presumably more physiological voltages, which in endosomes may be largely set by the similar voltage dependence of the acid-activated Cl^−^ channel ASOR/TMEM206 (*84*), suggests a GoF that is compatible with a dominant effect on the clinical outcome. The steep activation of WT ClC-6 transport at extreme voltages remains enigmatic as it is unclear under which circumstances these voltages might be achieved in vivo. Their relationship to previously reported small, almost instantaneous, ClC-6 currents (*11*), which share the voltage dependence with other CLCs, remains unclear.

The shift in voltage dependence with mutants affecting F317 and T520 suggested that they may cause neurological syndromes similar to that observed with p.Y553C (*4*). Strikingly, this prediction turned out to be correct when we identified a patient with a de novo p.T520A pathogenic variant (fig. S11A). The symptoms of this patient largely overlapped with those observed with the p.Y553C substitution (*4*). However, her symptoms appeared less severe, maybe related to the fact that both the shift in voltage dependence and gating kinetics of T520A were less pronounced than with Y553C.

Similar to Y553C, T520A induced the generation of giant lysosome-like vesicles in transfected cells. Likewise, their formation was prevented when inserting a *td* mutation that largely abolishes ClC-6 ion transport, and vesicle size was reduced when uncoupling Cl^−^ from H^+^ transport by an *unc* mutation (fig. S11B). Generation of large vesicles was also observed by another randomly selected mutant, which shifted the voltage dependence to the left, E550A. Hence, the generation of large vesicles can be blamed on increased 2Cl^−^/H^+^ exchange, H^+^ gradient-driven luminal Cl^−^ uptake, osmotic swelling, and inhibition of vesicle budding from “pressurized” vacuoles.

The present structure predicts several other residues as hotspots for disease-causing GoF mutations. Other mutations may result in LoF due to a shift of activation to even more positive voltages. Re-analysis of *CLCN6* variants previously identified in patients with mild NCL (*6*) revealed a similar shift to radically positive activation voltages (*16*). While the clinical symptoms of those heterozygous patients are congruent with the mild storage phenotype of homozygous *Clcn6*^−/−^ mice (*6*), heterozygous *Clcn6*^+/−^ mice lack central nervous system symptoms. Hence, these LoF mutations might cause a dominant negative effect by shifting the voltage dependence of WT subunits in ClC-6 WT/mutant dimers as observed with ClC-7 (*57*) and dominant ClC-1 mutants in human myotonia (*43*).

While the present ClC-6 structure closely resembles that of other CLC channels and exchangers, it revealed apparently ClC-6–specific interactions between CBS domains. These domains were also found to bind ATP. Both interactions modulate ClC-6 gating. The structure allowed us to identify key interactions between amino acids located in different helices and loops. Mutagenesis showed that these interactions are important for voltage-dependent gating, implying that this process involves conformational changes in various parts of the protein. Our analysis predicts hotspots for disease-causing mutations, one of which was confirmed by the identification of a de novo variant in a patient with severe neurological disease. Future clinical findings will reveal whether our prediction of other hotspots turns out to be correct. Our work not only advances our understanding of CLC gating in general but also has direct implications for human disease and possibly for the design of drugs aimed at their cure.

## MATERIALS AND METHODS

### Protein expression and purification

The cDNA encoding human ClC-6 was subcloned into the pEG BacMam expression vector with a C-terminal tandem twin Strep-tag. The resulting BacMam viruses were amplified to the second passage (P2) virus by using Sf9 cells (Invitrogen). P2 BacMam viruses of ClC-6 were transfected into human embryonic kidney (HEK) 293F cells (Thermo Fisher Scientific Inc.) when the cell density reached at 2 × 10^6^ cells per milliliter. Sodium butyrate (Sigma-Aldrich) was added to a final concentration of 10 mM, and cells were cultured for an additional 48 hours. Cells were then harvested by centrifugation at 3000*g*, and the cell pellet was resuspended in lysis buffer containing 150 mM NaCl, leupeptin (1 μg/ml), pepstatin (1.5 μg/ml), aprotinin (0.84 μg/ml), phenylmethylsulfonyl fluoride (0.3 mM), 20 mM Hepes (pH 7.4) and lysed by sonication for 5 min. Membranes were obtained by centrifugation at 100,000*g* for 1 hour. The pellet was resuspended with buffer containing 150 mM NaCl, 2 mM dithiothreitol (DTT), 0.1% (w/v) digitonin, and 20 mM Hepes (pH 7.4) for 2 hours with gentle rotation at 4°C, followed by an ultracentrifugation at 100,000*g* for 20 min to collect the supernatant. The supernatant was incubated with Strep-Tactin Sepharose (IBA Lifesciences) for 1 hour with gentle rotation at 4°C, and the resin was washed with a wash buffer containing 150 mM NaCl, 2 mM DTT, 0.1% (w/v) digitonin, and 20 mM Hepes (pH 7.4). Last, the ClC-6 protein was eluted with a wash buffer with the adding of 5 mM d-desthiobiotin (IBA Lifesciences). The protein sample was concentrated to a final volume of approximately 100 μl. The concentrated protein sample was further purified by size exclusion chromatography (Superpose 6 10/300 GL, GE Healthcare) with the elution buffer containing 150 mM NaCl, 0.1% (w/v) digitonin, and 20 mM Hepes (pH 7.4). The peak fraction for ClC-6 protein was collected for further cryo-EM study. The expression and purification protocol for ATP-bound ClC-6 and Y553C mutant were similar to apo–ClC-6 except that 2 mM ATP and 2 mM MgCl_2_ were added during the size exclusion chromatography to obtain the ATP-bound structure.

### Cryo-EM sample preparation and data acquisition

For samples of apo–ClC-6 proteins, 4 μl of purified protein at concentration of approximately 8 mg/ml was applied to glow-discharged Au 300-mesh R1.2/1.3 holey carbon grids (Quantifoil). The grids were plunged into liquid nitrogen-cooled liquid ethane for quick freezing with Vitrobot Mark IV (FEI). Cryo-grids were first screened on a Tecnai Arctica microscope (FEI) operated at 200 kV (equipped with a FEI Falcon II 4 k × 4 k camera). Data acquisition was performed with a Titan Krios microscope (FEI) operated at 300 kV with a Gatan K2 Summit detector equipped with a Gatan Imaging Filter (GIF) Quantum energy filter. Images were automatically recorded by SerialEM with a slit width of 20 eV on the energy filter. The detector in super-resolution mode at a nominal magnification of ×130,000, corresponding to a calibrated pixel size of 1.08 Å at object scale, and defocus range between 1.3 and 2.3 μm were used for data collection. Images were recorded for 5.6 s with an exposure time of 0.175 s per frame (total 32 frames per stack) (total of 32 frames per stack), corresponding a total dose of about 50 e/Å^2^. For samples of ATP-bound ClC-6 and Y553C mutant, data acquisition was performed with a Titan Krios microscope (FEI) operated at 300 kV with a Gatan K3 Summit detector equipped with a GIF Quantum energy filter, with a nominal magnification of ×105,000, corresponding to a calibrated pixel size of 0.8374 Å at object scale.

### Cryo-EM data processing

A total of 2850, 2340, and 3230 micrographs were collected for ClC-6, ATP-bound ClC-6, and Y553C mutant, respectively. To obtain summed micrographs with or without dose weighting, the datasets were further processed by subregion motion correction and dose weighting by MotinCor2. The contrast transfer function (CTF) parameters were estimated by CTFFIND4. Around 1,460,000, 1,238,000, and 1,840,000 particles of each data were manually picked and processed after 2D classification using RELION 3.0. The resulting 2D averages were used to generate initial templates for particle autopicking. Noise and bad particles were excluded by two rounds of 2D classifications. With three rounds of 3D classification, we obtained 182,000, 112,000, and 145,000 particles with good signal for ClC-6, ATP-bound ClC-6 and Y553C mutant, respectively. The local defocus parameters were refined using Gctf. The resultant particles were recentered and processed by autorefine with soft mask and C2 symmetry imposed using RELION 3.0 to increase resolution. The final density maps at the resolution of 3.5, 3.4, and 3.4 Å were obtained for ClC-6, ATP-bound ClC-6, and Y553C mutant, respectively. The resolutions were evaluated using ResMap and displayed in Chimera.

### Structural modeling and visualization

Models of full-length ClC-6 were predicted on I-TASSER server, followed by docking the models into the ClC-6 density map using Chimera. The frame was manually adjusted in COOT to acquire the atomic model of ClC-6. Sequence alignment and secondary structure prediction of ClC-6 were carried out for the assistance of model building. All models were refined against the density map using the real_space_refine module of PHENIX with secondary structure and geometry restraints. The final models were evaluated by MolProbity.

### Whole-cell patch-clamp recording and data analysis

The DNA encoding human ClC-6 was subcloned into the pcDNA3.1(−) vector with an N-terminal GFP-tag. Chinese hamster ovary (CHO) cells were cultured in Dulbecco’s modified Eagle’s medium (Thermo Fisher) supplemented with 10% fetal bovine serum (Gibco) and 1% penicillin-streptomycin solution under 5% CO_2_ at 37°C incubator. ClC-6 plasmids (2 μg/1.5 ml, into 35 mm–by–10 mm cell culture dish) were transfected into the CHO cell by using a Lipofectamine 3000 transfection kit (Invitrogen) according to the instructions of the manufacturer, and cells were cultured for additional 24 hours before patch-clamp experiments. The morphology of CHO cells overexpressing WT ClC-6 and some mutants (Fig. 6D) was observed and acquired under a fluorescence microscope (Leica, Germany).

All whole-cell patch-clamp recording on CHO was conducted with Axopatch-200B amplifier and Digidata 1440A digitizer (Molecular Devices). The pipettes were pulled with micropipette puller (P-97; SUTTER INSTRUMENT) and had a resistance of 3 to 5 megohm when filled with the solution containing 140 mM CsCl, 5 mM EGTA, 1 mM MgCl_2_, and 10 mM Hepes (pH was adjusted to 7.2 with CsOH). In most conditions, cells were bathed in the solution containing 150 mM NaCl, 6 mM KCl, 1 mM MgCl_2_, 1.5 mM CaCl_2_, 10 mM glucose, and 10 mM Hepes (pH was adjusted to 7.5 with NaOH). For the recording under high-external Cl^−^ environment, the bath solution was replaced by 210 mM NaCl, 6 mM KCl, 1 mM MgCl_2_, 1.5 mM CaCl_2_, 10 mM glucose, 10 mM Hepes and (pH 7.5). For the recording under low external Cl^−^ environment, partial NaCl was replaced by equimolar Na-aspartate to obtain the bath Cl^−^ concentrations of 51 and 11 mM. For the recording under varied external pH, solutions were buffered with 10 mM Hepes, MES, and tris as appropriate. The bath temperature was controlled by using automatic temperature controller with negative feedback (Warner, TC-344C), and the bath temperature was set at 25° ± 0.5°C in normal condition. Whole-cell currents were evoked by clamping the cells for 0.8 s to voltages between −100 mV and +160 mV in 20-mV increments and then followed by a repolarizing step to −30 mV for 0.4 s. The series resistant was compensated before forming tight patch seal. Further series resistance and capacitance compensation were applied after forming whole-cell recording. Liquid junction potentials were less than 2 mV that were calculated using JPCalc software (*85*). The voltage-evoked currents were fitted and analyzed with the software of Clampfit 10.2 (Molecular Devices).

To quantify the activation/deactivation kinetics, the first 300-ms activation process of depolarization-triggered currents and the first 100-ms decaying process of tail currents were fitted to a single exponential function

$$I={Ae}^{-t/\tau }+C$$ (1)

where *I* is the current amplitude, *t* is the time, A is the amplitude constant, τ is the time constant.

Analysis of the temperature-dependent activation kinetics of ClC-6 was performed as described previously (*22*, *23*). Briefly, the value of τ_activation_ at varied temperature was fitted by the Arrhenius equation

$$\ln k=lnA-E_{a}/RT$$ (2)

where *k* is a rate constant (1/τ_activation_) at the absolute temperature *T*, *R* is the gas constant, *A* is a constant factor. By measuring the slope of the Arrhenius plots (ln*k* versus 1/*T*), we could calculate the value of the activation energy (*E*_a_). The enthalpic component (∆*H*^‡^) was calculated as following

$$\text{Δ}H^{‡}=E_{a}-RT$$ (3)

The value of the temperature coefficient (*Q*_10_) was calculated from the Arrhenius plots according to *Q*_10_ = *k*(*T* + 10 K)/*k*(*T*), for *T* = 293 K.

To approximately quantify the shifting of voltage dependence, current densities were divided by the respective voltage driving force and plotted as a function of voltage. This procedure gives a relative macroscopic conductance that is proportional to the “open probability” (*p*_open_) of the gated transporter. In the most left-shifted mutant (Y553A), the conductance reached a plateau at the most positive potential, presumably since *p*_open_ approached 1. For the more right-shifted WT and mutants, where no plateau was reached in the experimental voltage range, we took the maximum value obtained from the Boltzmann fit of the left-shifted mutant (Y553A) as upper limit for fitting. These fits gave an “apparent *V*_1/2_”, i.e., the voltage where *p*_open_ = 0.5.

### Immunofluorescence of transfected cells

Immunofluorescent staining of ClC-6 of transfected HeLa cells using an anti–ClC-6 antibody (*11*) and anti-LAMP1 (H4A3, Abcam) was performed as described (*4*). HeLa cells were transfected using FuGENE 2 days before immunostaining at 1:3 DNA:reagent ratio. Cells were fixed with 4% paraformaldehyde in phosphate-buffered saline (PBS) for 15 min. Then, cells were incubated with 25 mM glycine in PBS for 5 min and permeabilized with 0.05% saponin in PBS for 10 min. Antibodies and 4′,6-diamidino-2-phenylindole dye were applied in PBS with 0.05% saponin and 3% bovine serum albumin. Cells transfected with untagged ClC-6 were co-immunostained with a rabbit antibody against the ClC-6 C terminus [6C3 (*11*)] and a mouse anti-LAMP1 antibody (H4A3, Abcam). Subsequently, cells were incubated with goat-derived secondary antibodies conjugated to Alexa Fluor 488 or Alexa Fluor 555 (Molecular Probes). Confocal images were obtained with an LSM880 laser scanning confocal microscope with a 63 × 1.4 numerical aperture oil immersion lens (Zeiss).

### Subjects

This project was approved by the local Institutional Ethical Committee of the Ospedale Pediatrico Bambino Gesù (1702_OPBG_2018), Rome, and the Ethics Committee of the Hamburg Medical Chamber (PV3802). Subject was analyzed in the frame of a research project dedicated to undiagnosed patients. Clinical data and biological material were collected, stored, and used according to procedures in accordance with the ethical standards of the Declaration of Helsinki protocols, with signed informed consents from the participating families.

### Statistical analysis

Analysis of currents and curve fitting used Clampfit 10.2. Results were expressed as means ± SEM. All statistical analyses were performed using SPSS 20.0. Unpaired Student’s *t* test, one-way analysis of variance (ANOVA), or two-way ANOVA with post hoc Bonferroni tests were performed for the difference analysis between groups. The number of tested cells/transfections in each group and the statistical methods were indicated in individual figure legends and summarized in a supplementary table (table S3). The results were evaluated to be statistically significant when *P* < 0.05, *P* < 0.01, or *P* < 0.001.

## Supplementary Materials

### This PDF file includes:

Case Report Figs. S1 to S11 Tables S1 to S3
