# Supplementary material for: Molecular basis of ClC-6 function and its impairment in human disease
Source: Sci Adv. 2023 Oct 13;9(41):eadg4479. doi: 10.1126/sciadv.adg4479 (PMC10575590; doi:10.1126/sciadv.adg4479)
Supplement: Supplementary file 1 — Case Report Figs. S1 to S11 Tables S1 to S3 [file sciadv.adg4479_sm.pdf]

Science Advances
AAAS

Supplementary Materials for

Molecular basis of ClC-6 function and its impairment in human disease

Bing Zhang *et al.*

Corresponding author: marco.tartaglia@opbg.net; liyang@simm.ac.cn; jentsch@fmp-berlin.de; maojunyang@tsinghua.edu.cn; liuzhiqiang@51mch.com

Sci. Adv. **9**, eadg4479 (2023)
DOI: 10.1126/sciadv.adg4479

This PDF file includes:

Case Report
Figs. S1 to S11
Tables S1 to S3

Case Report

The proband is the third, and only affected child of a non-consanguineous couple without any significant medical history. She was born at 36 weeks of gestation after a non-complicated dizygotic twin pregnancy. At 14 months, she was referred to a neuropediatrician for developmental delay compared to her twin-sister. Her clinical examination revealed a good contact, global hypotonia, absent deep tendon reflexes. The parents had observed some behavioral arrest but interictal EEG was normal.

Since 16 months of age, she presented recurrent paroxysmal episodes characterized by hypopnea/apnea, requiring several periods of invasive ventilation, loss of consciousness and sometimes tonic or myoclonic movements of the upper limbs, occurring generally during febrile infection, suspected of being epileptic seizures. During the first episode, initial workup included cerebral and medullar MRI showing infra- and supratentorial symmetrical T2 hyperintensity, frontal white matter hypomyelination as well as T2 hyperintensity and apparent swelling in the spinal cord at cervical level. Several extubation attempts failed due to persistent hypopnea with significant hypercapnia. Her condition improved following the initiation of corticotherapy that was initiated because of the swelling spinal cord. Successful extubation could be achieved after 5 weeks but the patient remained dependent on non-invasive ventilation during sleep due to persistent hypopnea. An epileptic origin to her acute episodes was suspected, and an anti-seizure medication (ASM) was given, which progressively escalated over the years. Numerous interictal EEG showed slow wave activity compatible with mild to moderate encephalopathy but no epileptic activity. Ictal EEG was recorded twice, both during an acute episode of behavioral arrest, loss of consciousness, and apnea. The first one did not show any epileptic activity. The second one showed a prolonged focal seizure characterized by rhythmic 1Hz spike-and-waves activity in the left temporal region during ten minutes, that stopped after the administration of benzodiazepine and was associated with a recuperation of spontaneous breathing and consciousness. Therefore, we concluded that the patient presented repeated acute episodes with apnea and loss of consciousness of epileptic and non-epileptic origin.

During a 3-year period, we repeated brain and medullar MRI during several paroxysmal

episodes and did not evidence any abnormalities in the DWI and ADC images, as seen in other patients with *CLCN6* mutations. Nevertheless, we evidenced a diffuse supratentorial demyelination on T2 WI. Metabolic workup as well as initial whole exome sequencing was inconclusive. Because of areflexia, EMG and conduction nerve velocities were performed twice and inconclusive.

The patient also presented bilateral cataracts, as well as exotropia. Cataracts where first observed at 1 year of age, confirmed at age 3, but where not observed again during ophthalmologic examination at age 5. At 5 years, after a paroxysmal episode of loss of consciousness and apnea, visual impairment secondary to cortical blindness was diagnosed, with global hypometabolism more distinct in the bilateral occipital cortex on brain FDG-PET scan. The administration of steroids improved her vision but she never regained the vision that she had before that episode. Indeed, she presents a visual acuity of 1/20 and 1/10 for the right and the left eye respectively. Furthermore, she presented a hypermetabolism in the putamen bilaterally and a hypometabolism in the cerebellum and both thalami.

Currently she is 6 years old, is able to walk with support, and shows some progress in expressive language. She shows some feeding difficulties with poor swallowing.

Whole genome sequencing performed in 2021 identified a *de novo CLCN6* variant (c.1558A>G, p.T520A, NM_001286.3; not reported in gnomAD, CADD: 24.5 (deleterious); REVEL: deleterious (moderate) (0.84), Mutation Taster: deleterious (1), BayesDel: deleterious (moderate) (0.38), MetaLR: deleterious (0.79), SIFT: uncertain (0.03), FATHMM: uncertain (−3.26), GenoCanyon: deleterious (1); fitCons: deleterious (0.67); ACMG criteria: likely pathogenic (PS2,PM2, PP3)), which lead to a tentative diagnosis of childhood-onset neurodegeneration with hypotonia, respiratory insufficiency, and brain imaging abnormalities(OMIM: 619173).

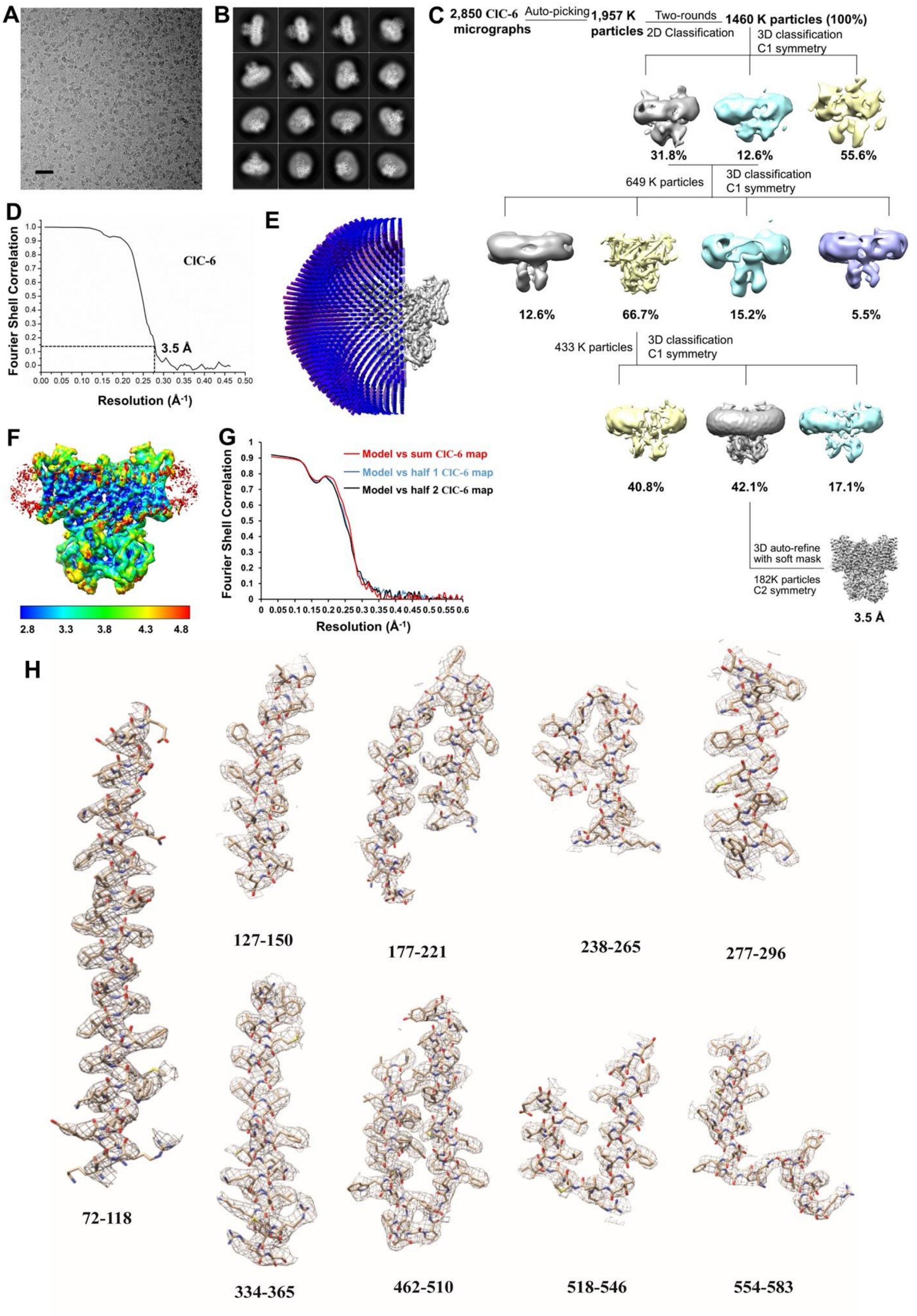

Fig. S1. Structure determination and reconstruction of ClC-6. (**A**) Representative cryo-EM micrograph of ClC-6 in digitonin buffer. The scale bar represents 50nm. (**B**) 2D class averages of the ClC-6 sample in digitonin buffer. (**C**) Flow chart of the ClC-6 data

processing by Relion software. (**D**) Gold-standard Fourier Shell correlation (FSC) curve of ClC-6 after 3D refinement. The resolution estimation was based on the criterion of FSC 0.143 cutoff. (**E**) Angular distribution of the ClC-6 final reconstruction. (**F**) Local resolution map of the ClC-6 after the final 3D density map. (**G**) Cross-validation of the atomic model with the summed map and the half maps of ClC-6. (**H**) Representative cryo-EM densities of ClC-6.

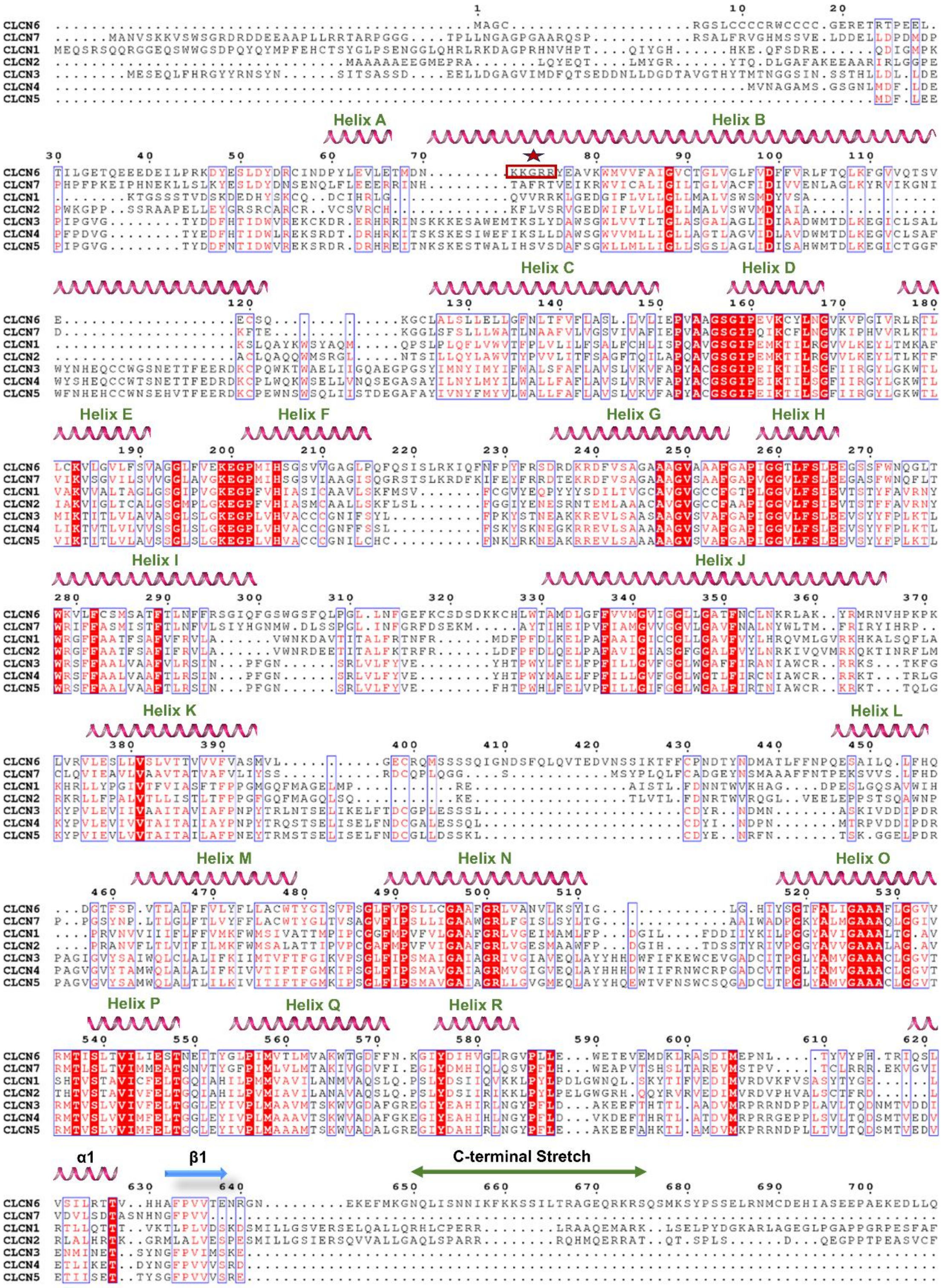
CLCN6
CLCN7
CLCN1
CLCN2
CLCN3
CLCN4
CLCN5
Helix A
Helix B
Helix C
Helix D
Helix E
Helix F
Helix G
Helix H
Helix I
Helix J
Helix K
Helix L
Helix M
Helix N
Helix O
Helix P
Helix Q
Helix R
α1
β1
C-terminal Stretch

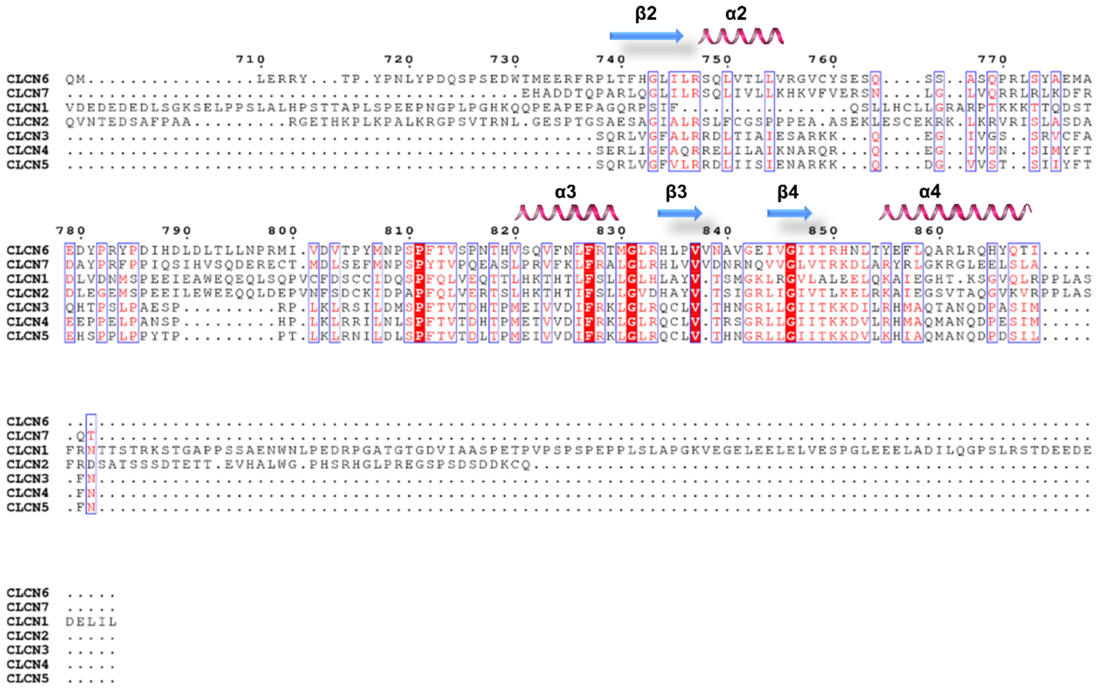

Fig. S2. Sequence alignment of human ClC-1 to ClC-7. The KKGRR stretch (on helix B) is highlighted by a red box and a star. The C-terminal stretch between the CBS domains (residues 650–676) which protrudes into the opposite monomer is highlighted with arrowed line.

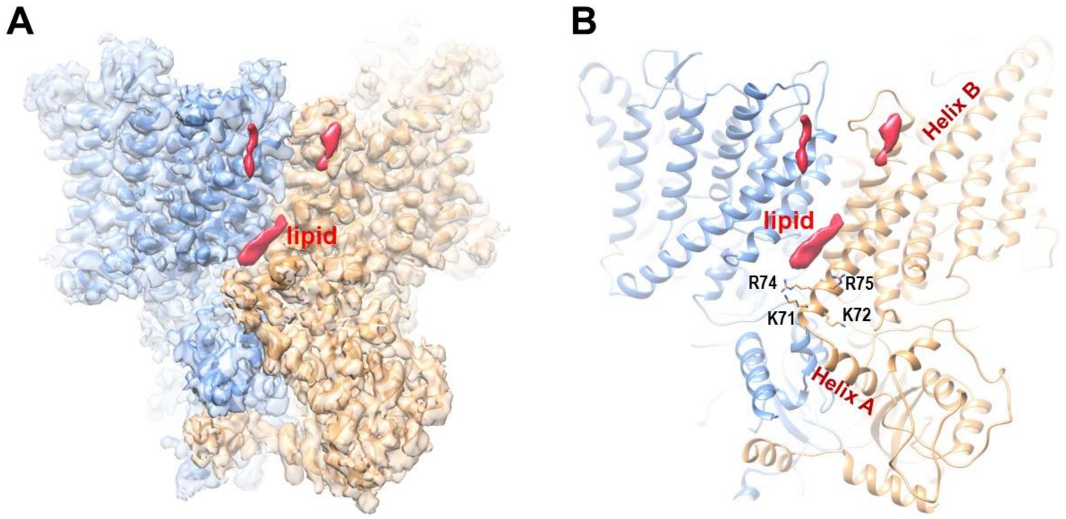

Fig. S3. Cryo-EM map of the ClC-6 viewed from side. (**A** and **B**) ClC-6 structure with each subunit color coded (blue and brown). The KKGRR region (on helix B) were shown as sticks and the densities for the lipids were colored as red.

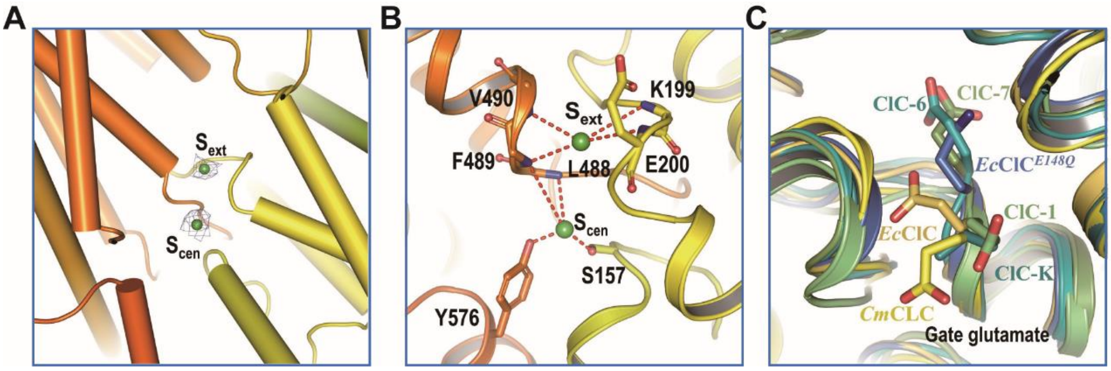

Fig. S4. Ion translocation pathway of ClC-6. (**A**) Stereo view of the ion-binding sites. Cl^- binding sites (S_{ext} and S_{cen}) are shown as blue spheres. (**B**) The coordinating residues for S_{ext} and S_{cen}. (**C**) The structural superimposition of ClC-6 with ClC-1, ClC-7, *Ec*ClC^{WT}, *Ec*ClC^{E148Q} and *Cyanidioschyzon merolae* (*Cm*ClC) at the E_{gate} position.

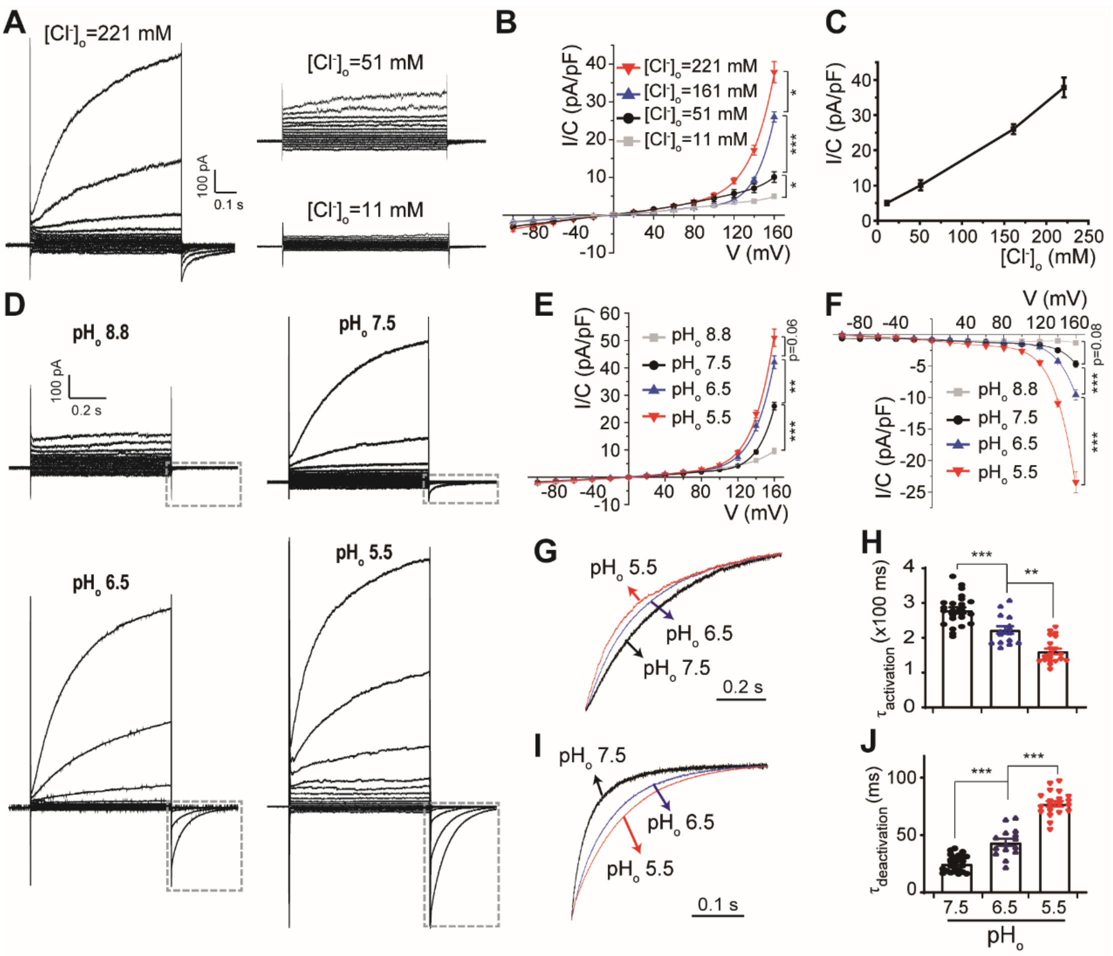

Fig. S5. Chloride- and Proton-dependent activity of ClC-6. (**A** to **C**) The influence of external Cl^- level on the currents of WT ClC-6 (n=8/2 for $[Cl^-]_o$=221 mM; n=9/3 for $[Cl^-]_o$=51 mM; n=7/2 for $[Cl^-]_o$=11 mM). The representative current traces (A) and I-V

curves (B) were shown, and the current densities at +160 mV were compared. The current densities at +160 mV were plotted with external Cl^- concentration (C). (**D** to **F**) Representative current traces and the I-V curves of stepped-voltage triggered currents and tail currents of WT ClC-6 obtained under different external proton level (pH_o: 8.8, 7.5, 6.5, 5.5; the corresponding n=13/3, 24/7, 15/4, 20/5), the current densities at +160 mV were compared. Extracellular alkalinization (pH_o 8.8) resulted in undetectable tail currents, whereas extracellular acidification (pH_o 6.5 or 5.5) dramatically increased the visible number and the magnitudes of the tail current. (**G** to **J**) The current and tail current curves at +160 mV were normalized to compare the activation and deactivation rate under different external proton level (pH_o: 7.5, 6.5, 5.5). The external acidification decreases the value of $\tau_{activation}$ but increases the value of $\tau_{deactivation}$, suggesting a dual regulatory effect for the activation and deactivation of ClC-6 by pH_o. Data were represented as means ± SEM, one-way ANOVA with post hoc Bonferroni tests (B, E, F, H, J) were performed, $^{*}P<0.05$, $^{**}P<0.01$ and $^{***}P<0.001$.

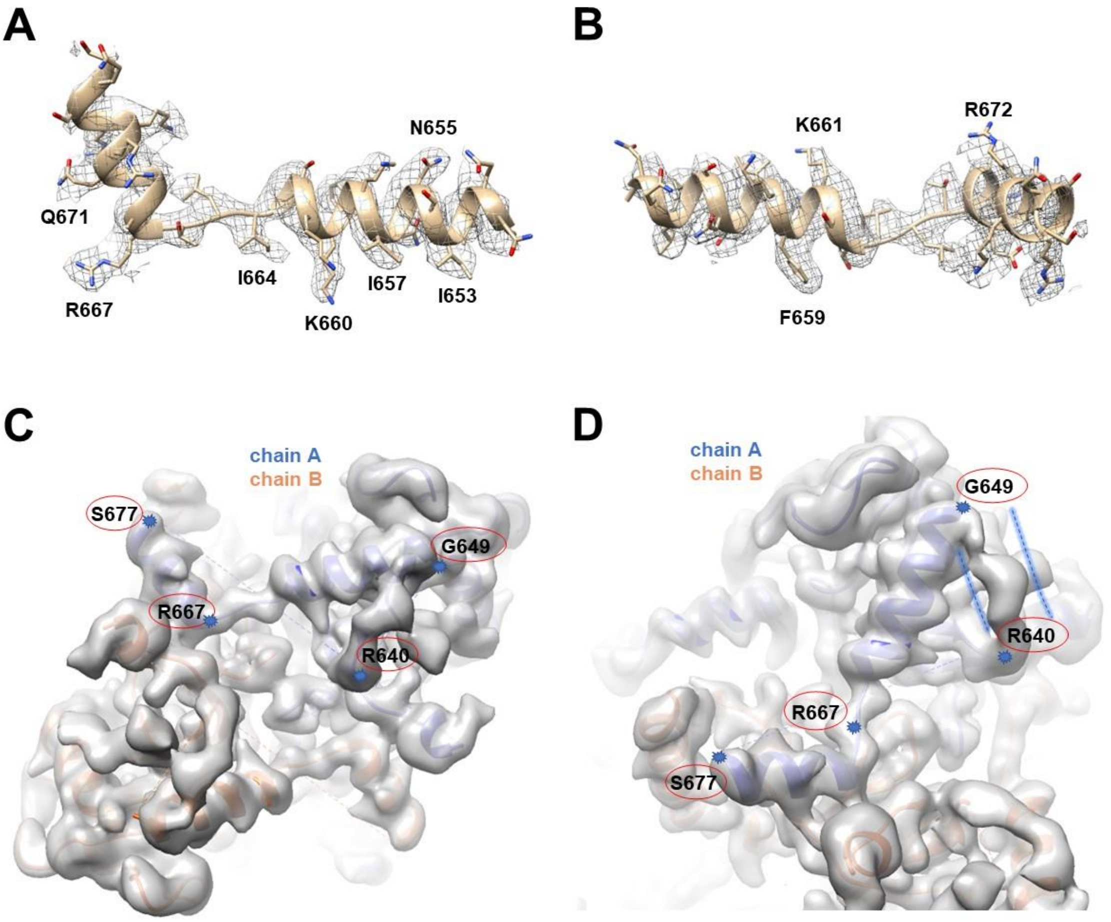

Fig. S6. The helix-turn-helix structure (649-677) in ClC-6. (**A** and **B**) Cryo-EM density map of the helix-turn-helix structure (649-677) in ClC-6 with bulky side chain residues labeled. (**C** and **D**) The un-postprocessed density map of the helix-turn-helix region in ClC-6. The blue dotted line indicates the continuous linker and the orientation of helix-turn-helix from one protomer to another protomer.

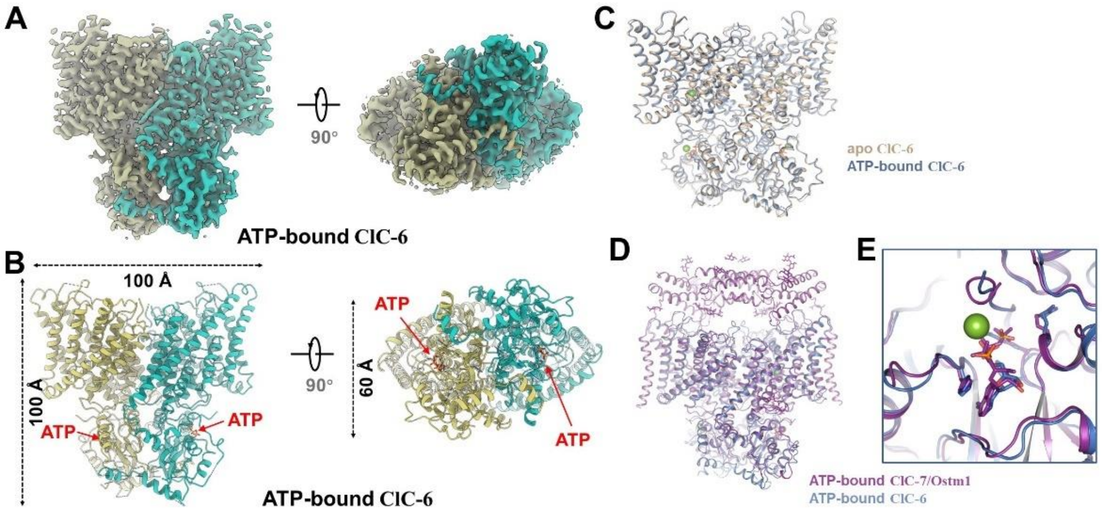

Fig. S7. Cryo-EM structure of ATP-bound ClC-6. (**A** and **B**) Overall structure of ClC-6 in complex with ATP. The ATP molecules were shown as red sticks. (**C**) Structural superimposition of apo ClC-6 with ATP-bound ClC-6. (**D** and **E**) Structural superimposition of ATP-bound ClC-6 and ATP-bound ClC-7/Ostm1 (PDB:7JM7). The magnified view for ATP binding pocket were shown in panel (E).

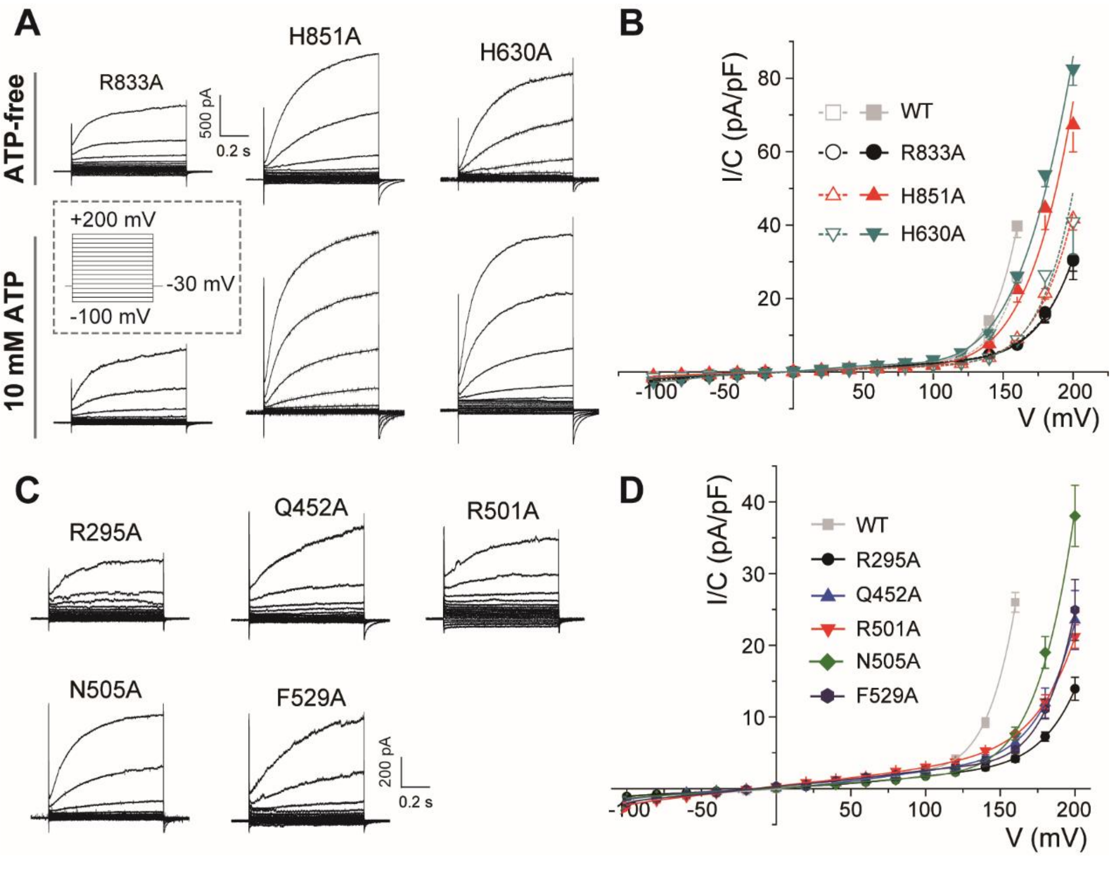

Fig. S8. The electrophysiological performance of mutants under extremely high

voltage. (**A** and **B**) Representative current traces and I-V curves of three mutants, including R833A, H851A and H630A, under extremely high voltage (up to 200 mV) without or with 10 mM intracellular ATP. The electrophysiological protocol is inserted in panel (A, middle left): cells were hold at −30 mV, and then the voltage was clamped from −100 to +200 mV in 0.8 s steps of 20 mV, then stepping down to −30 mV. (**C** and **D**) Representative current traces and I-V curves of five mutants, including R295A, Q452A, R501A, N505A and F529A, under extreme high voltage (up to 200 mV).

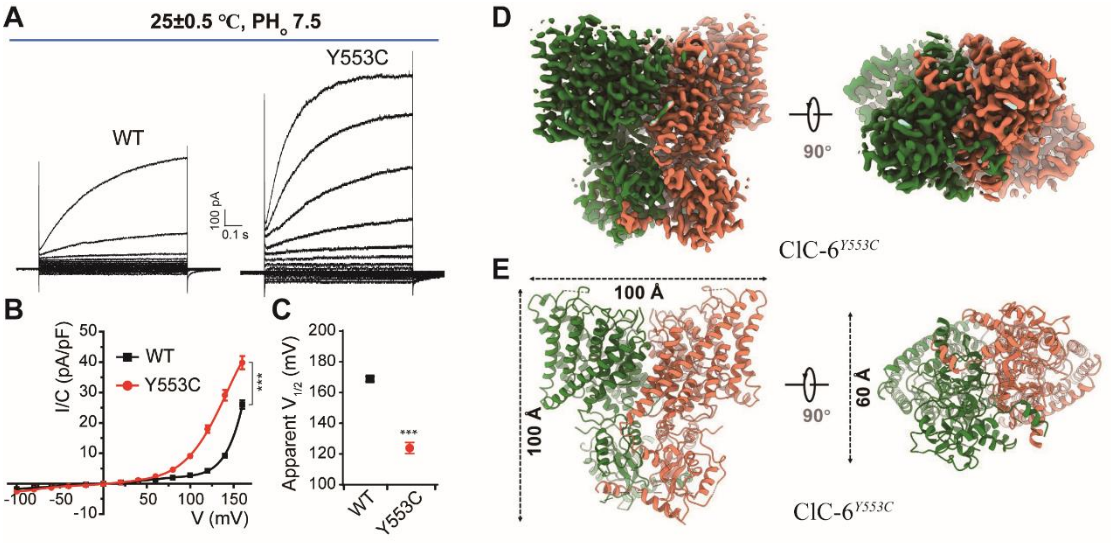

Fig. S9. Electrophysiological characteristics and cryo-EM structure of Y553C mutant. (**A** to **C**) The electrophysiological performance of Y553C mutant. Representative current traces and I-V curves of WT ClC-6 and Y553C measured under normal (25±0.5 °C, pH_o 7.5; n=13/4 for Y553C) condition (A-B). Apparent $V_{1/2}$ were compared between WT ClC-6 and Y553C mutant (C). (**D** and **E**) Cryo-EM density map and overall structure model of ClC-6^{Y553C} mutant. Data were represented as means ± SEM, unpaired student's *t*-tests (B, C) were performed, ****P*<0.001.

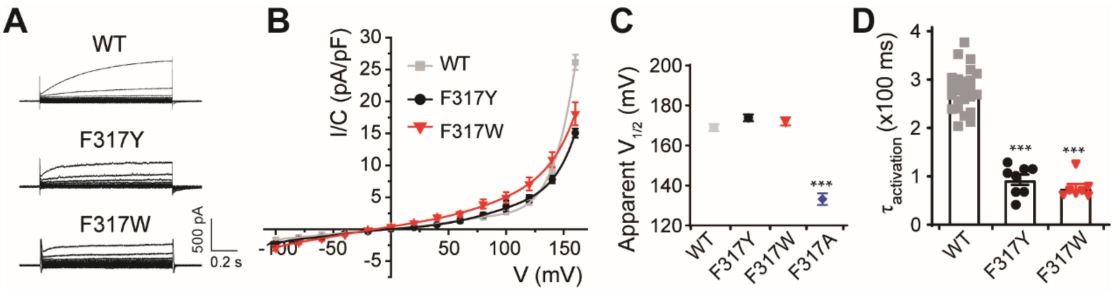

Fig. S10. Effects of F317Y and F317W on the voltage dependence and activation kinetics of ClC-6. (**A** and **B**) Representative current traces and I-V curves of WT ClC-6 and mutants including F317Y (n=8/2) and F317W (n=8/2). (**C**) Apparent $V_{1/2}$ were compared between groups shown in panel (A and B). (**D**) The value of $\tau_{activation}$ at 160 mV were compared between groups shown in panel (A and B). Data were represented as means ± SEM, one-way ANOVA with post hoc Bonferroni tests (C, D) were performed, $^{***}P$<0.001 (versus WT ClC-6).

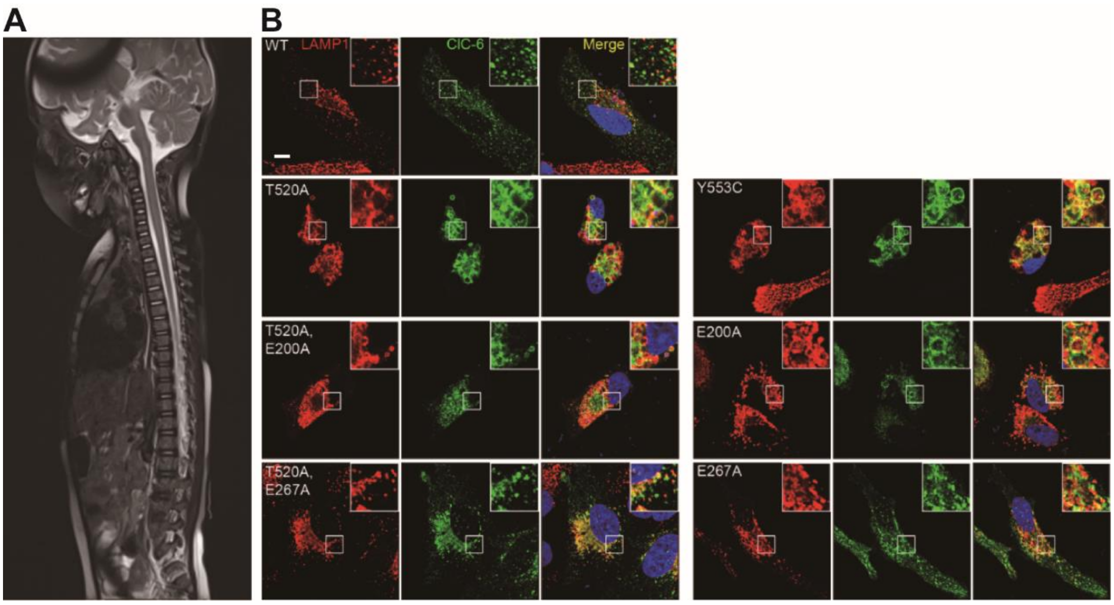

Fig. S11. The clinical and cell biological performance of pathogenic ClC-6 variants. (**A**) MRI feature of the subject with *de novo* c.1558A>G missense substitution (p.T520A) in *CLCN6*. MRI scan at age 16 months showing T2 hyperintensity from the pons of the brainstem to the C6 vertebra giving evidence of a swelling in the brainstem and the cervical cord. (**B**) Generation of giant LAMP1-positive vacuoles by disease-causing mutants. Co-immunostaining of overexpressed ClC-6 and endogenous LAMP1 in HeLa cells. Like Y553C, T520A overexpression causes formation of giant LAMP1-positive vesicles. The

formation of giant vesicles is dependent on the ion transport of the mutant, because it is largely suppressed by inserting the uncoupling ‘gating glutamate’ mutation E200A, which converts ClC-6 into a pure Cl^- conductance, or almost completely abolished by the transport-deficient E267A ‘proton glutamate’ mutation. The scale bar represents 10μm.

Table S1. Summary of clinical features of the subject with *de novo* p.T520A substitution in *CLCN6*.

**Basic information**	
Gene	*CLCN6*
Mutation (NM_001286.3)	c.1558A>G (p.T520A)
Origin	*de novo*
Ethnic background	South-American
Sex	female
**Clinical features**	
Pregnancy	Uncomplicated dizygotic twin pregnancy
Birth at (weeks of gestation)	36 (iterative c-section for twin pregnancy)
Birth weight (g) (centile, z-score)	2500 g (41%, −0.23)
Birth length cm (centile, z-score)	45 cm (28%,−0.57)
OFC birth cm (centile, z-score)	unknown
**Last examination**	
Age	6 year, 3 months, alive
Weight kg (centile, z-score)	21.6 kg (60%, 0.24)
Height cm (centile, z-score)	104 cm (1%, −2.21)
BMI (centile, z-score)	19.4 (96%, 1.8)
OFC cm (centile, z-score)	48.6 cm
**Neurological features**	
Global developmental delay	global development delay; developmental regression first noted at 12 months
Motor development	sitting unsupported at 2 years; walking with aid since age 3; unsupported walking not acquired
Speech impairment	articulates about 20 different words; does some 2-3 words phrases
Muscular hypotonia	generalized hypotonia
Movement disorder	ataxic gait
Seizures	focal temporal epileptiform activity observed after one paroxysmic event at age 5
EEG features	excessive slow wave activity predominantly in anterior regions (present in all EEGs between age 1 and 6); focal left temporo-central spike-and-wave discharges (clinically associated with nystagmus and clonic movements of the right arm, resolved after one dose of Midazolam, present during one episode at age 5)
MRI scan	08/2017: infra- and supratentorial bilateral T2 hyperintensity; frontal white matter hypomyelination; spinal T2 hyperintensity and swelling<br>11/2021: delayed myelination and global white matter

	hypotrophy; thin corpus callosum; peritrigonal FLAIR hyperintensity
Neurogenic bladder	N
Abnormality of temperature regulation	episodes occurring generally during febrile infection; temperature instability noted during first event
**Other clinical findings**	
Cardiovascular system abnormalities	none (normal echocardiography in 2021)
Hearing abnormalities	none
Vision abnormalities	bilateral cataracts; visual impairment caused by optical atrophy and cortical blindness; nystagmus
Abnormalities of the respirator system	chronic respiratory insufficiency since 16 months of age; requiring intermittent non-invasive ventilation during sleep; recurrent episodes of apnea/hypopnea
Abnormalities of the skin	none
Feeding difficulties	yes (poor swallowing)
Craniofacial features	prominent forehead; slight hypotelorism; upslanting palpebral fissures
Other molecular findings	none
**Electrophysiology studies**	
VEPs	2017: normal<br>03/2021: normal<br>11/2021: deterioration of cortical visual responses with low amplitudes
ERG	NA
BAEPs	2017: indirect signs of transmissional auditory deficit<br>03/2021: normal<br>11/2021: microphonic cochlear potential inhibition deficit
Skin biopsy	normal respiratory chain analysis on skin fibroblasts
Muscle biopsy	slight to moderate lipid overload; slight muscular atrophy and variability in muscle fiber diameter
Nerve conduction studies	2021: normal
**Biochemistry**	
Copper (µg/dL)	NA
Ceruloplasmin (mg/dL)	NA
Copper excretion (µg/24h)	NA
VLCFA	Normal
Blood Lactate (mM)	18.2 mg/dL
CSF Lactate (mM)	13 mg/dL
Plasma Alanine (µmol/L)	338 µmol/L
Plasma Creatine (µmol/L)	0.26 mg/dL

Table S2. Cryo-EM data collection, refinement and validation statistics

	Apo-ClC-6	ATP-bound ClC-6	ClC-6^{Y553C}
**Data collection and processing**			
Magnification	130,000	105,000	105,000
Voltage (kV)	300	300	300
Electron exposure (e^-/Å^2)	50	50	50
Defocus range (μm)	-1.0 ~ -2.5	-1.0 ~ -2.5	-1.0 ~ -2.5
Pixel size (Å)	1.08	0.8374	0.8374
Software	RELION	RELION	RELION
Symmetry imposed	C2	C2	C2
Initial particle images (no.)	1,957,483	1,431,564	2,231,564
Final particles images (no.)	182,338	112,377	145,232
Map resolution (Å)	3.5	3.4	3.4
FSC threshold	0.143	0.143	0.143
Local map resolution range (Å)	4.8-2.8	4.8-2.8	4.8-2.8
**Refinement**			
Software	PHENIX 1.14	PHENIX1.14	PHENIX1.14
Model resolution (Å)	3.5/3.6	3.4/3.5	3.4/3.4
FSC threshold	0.143/0.5	0.143/0.5	0.143/0.5
Map sharpening *B* factor	130.2	149.1	175.1
Model composition			
Non-hydrogen atoms	11448	11476	11396
Protein residues	1466	1428	1460
Ligand	4	8	8
B factors (Å^2)			
Protein	88.32	30.16	27.84
Ligand	30.00	23.17	20.57
R.m.s deviations			
Bond length (Å)	0.003	0.004	0.003
Bond angles (°)	0.588	0.628	0.556
Validation			
MolProbity score	1.45	1.50	1.41
Clashscore	4.91	6.25	6.87
Poor rotamers (%)	0	0	0
Ramachandran plot			
Favored (%)	96.82	97.16	97.85
Allowed (%)	3.18	2.84	2.15
Disallowed (%)	0	0	0

Table S3. Summary for the statistic details of electrophysiological results

Fig 2D	Cells/Batches	Mean±SE (pA/pF)	Statistics	Sign. (p value)	Sign		
**Blank cell**	9/4	3.13±0.16	unpaired Student's t-tests				
**WT**	24/7	25.99±1.36		<0.001 (vs. Blank cell)			
**Fig 2F**	**Cells/Batches**	**Mean±SEM (ms)**	**Statistics**	**Sign. (p value)**	**Sign**		
**WT 17℃**	16/4	974.36±60.19	one-way ANOVA	<0.001 (vs. 23℃)	***		
**WT 23℃**	12/3	339.11±28.61					
**WT 29℃**	11/3	119.04±9.96		0.002 (vs. 23℃)	**		
**WT 34℃**	13/3	81.91±8.77		<0.001 (vs. 23℃)	***		
**Fig 3D**	**Cells/Batches**	**Mean±SEM (ms)**	**Statistics**	**Sign. (p value)**	**Sign**		
**WT**	24/7	280.09±9.93	one-way ANOVA				
**E266A**	10/3	35.98±2.47		<0.001 (vs. WT)	***		
**Q274A**	9/3	45.37±3.76		<0.001 (vs. WT)	***		
**D52A**	9/3	94.43±12.28		<0.001 (vs. WT)	***		
**Y53A**	9/3	84.41±14.68		<0.001 (vs. WT)	***		
**D54A**	10/3	111.57±4.73		<0.001 (vs. WT)	***		
**N167A**	12/3	37.31±2.89		<0.001 (vs. WT)	***		
**D240A**	12/3	35.36±4.01		<0.001 (vs. WT)	***		
**R828A**	12/3	22.31±1.29		<0.001 (vs. WT)	***		
**Fig 3G**	**Cells/Batches**	**Mean±SEM (ms)**	**Statistics**	**Sign. (p value)**	**Sign**		
**WT**	24/7	280.09±9.93					

**Δ650-674**		10/3	143.15±9.26	one-way ANOVA	<0.001 (vs. WT)	***		
**R667A**		7/2	175.35±19.95		<0.001 (vs. WT)	***		
**R674A**		8/2	203.59±14.42		<0.001 (vs. WT)	***		
**Fig 4E**		**Cells/Batches**	**Mean±SEM (pA/pF)**	**Statistics**	**Sign. (p value)**	**Sign**		
**WT**	**ATP-free**	24/7	25.99±1.36	unpaired Student's t-tests				
	**10 mM ATP**	9/3	39.74±3.13		<0.001 (vs. ATP-free)	***		
**Fig 4H**		**Cells/Batches**	**Mean±SEM (mV)**	**Statistics**	**Sign. (p value)**	**Sign**	**Sign. (p value)**	**Sign**
**ATP-free**	**WT**	24/7	168.93±1.47	two-way ANOVA				
	**R833A**	11/3	209.75±3.5		<0.001 (vs. WT, ATP-free)	***		
	**H851A**	11/3	197.16±2.29		<0.001 (vs. WT, ATP-free)	***		
	**H630A**	10/3	195.79±3.28		<0.001 (vs. WT, ATP-free)	***		
**10mM ATP**	**WT**	9/3	152.91±1.47				0.002 (vs. ATP-free)	##
	**R833A**	12/3	207.34±4.35		<0.001 (vs. WT, 10 mM ATP)	***	0.996 (vs. ATP-free)	n.s.
	**H851A**	9/3	178.91±4.16		<0.001 (vs. WT, 10 mM ATP)	***	0.002 (vs. ATP-free)	##
	**H630A**	8/2	176.1±4.41		<0.001 (vs. WT, 10 mM ATP)	***	0.013 (vs. ATP-free)	#
**Fig 5D**		**Cells/Batches**	**Mean±SEM (mV)**	**Statistics**	**Sign. (p value)**	**Sign**		
**WT**		24/7	168.93±1.47	one-way ANOVA				
**Y553A**		11/3	99.44±2.51		<0.001 (vs. WT)	***		
**F317A**		12/3	133.21±2.56		<0.001 (vs. WT)	***		

**T520A**		7/3	146.72±2.95		<0.001 (vs. WT)	***		
**Fig 5E**			**Mean±SEM (ms)**	**Statistics**	**Sign. (p value)**	**Sign**		
**120 mV**	**WT**	24/7		one-way ANOVA				
	**Y553A**	11/3	223.69±20.35					
	**F317A**	12/3	40.76±2.76		<0.001 (vs. Y553A, T520A)	***		
	**T520A**	7/3	496.23±58.34					
**140 mV**	**WT**	24/7	433.26±35.56	one-way ANOVA				
	**Y553A**	11/3	149.24±9.35					
	**F317A**	12/3	35.97±2.32		<0.001 (vs. WT, Y553A, T520A)	***		
	**T520A**	7/3	248.91±20.89					
**160 mV**	**WT**	24/7	280.09±9.93	one-way ANOVA				
	**Y553A**	11/3	114.42±7.13					
	**F317A**	12/3	33.18±1.85		<0.001 (vs. WT, Y553A, T520A)	***		
	**T520A**	7/3	159.33±17.65					
**Fig 5H**		**Cells/Batches**	**Mean±SEM (mV)**	**Statistics**	**Sign. (p value)**	**Sign**	**Sig. (p value)**	**Sign**
**WT**		24/7	168.93±1.47	one-way ANOVA				
**Y553W**		6/2	165.33±2.49					
**Y553F**		11/3	163.3±1.96					
**Y553C**		13/4	123.9±3.14		<0.001 (vs. WT)	***		
**Y553A**		11/3	99.44±2.51		<0.001 (vs. WT)	***	0.000 (vs. Y553C)	###
**Fig 6C**		**Cells/Batches**	**Mean±SEM (mV)**	**Statistics**	**Sign. (p value)**	**Sign**		

**WT**		24/7	168.93±1.47	one-way ANOVA				
**Y553A**		11/3	99.44±2.51		<0.001 (vs. WT)	***		
**E550A**		11/3	117.42±2.73		<0.001 (vs. WT)	***		
**N549A**		11/3	143.269±2.13		<0.001 (vs. WT)	***		
**<u>Fig 6E</u>**		**Cells/Batches**	**Mean±SEM (ms)**	**Statistics**	**Sign. (p value)**	**Sign**		
**120 mV**	**Y553A**	11/3	223.69±20.35	one-way ANOVA				
	**E550A**	11/3	208.93±23.22					
	**N549A**	11/3	64.66±8.16		<0.001 (vs. Y553A, E550A)	***		
**140 mV**	**Y553A**	11/3	149.24±9.35	one-way ANOVA				
	**E550A**	11/3	132.42±12.14					
	**N549A**	11/3	48.59±6.96		<0.001 (vs. Y553A, E550A)	***		
**160 mV**	**Y553A**	11/3	114.42±7.13	one-way ANOVA				
	**E550A**	11/3	92.48±5.90					
	**N549A**	11/3	36.72256		<0.001 (vs. Y553A, E550A)	***		
**<u>Fig 7D</u>**		**Cells/Batches**	**Mean±SEM (ms)**	**Statistics**	**Sign. (p value)**	**Sign**		
**WT**		24/7	280.09±9.93	one-way ANOVA				
**L311A**		9/3	45.84±3.71		<0.001 (vs. WT)	***		
**L312A**		9/3	46.52±7.91		<0.001 (vs. WT)	***		
**F314A**		9/3	40.38±4.05		<0.001 (vs. WT)	***		
**F454A**		8/2	71.98±7.29		<0.001 (vs. WT)	***		
**H455A**		9/3	40.87±5.10		<0.001 (vs. WT)	***		
**<u>Fig 8D</u>**		**Cells/Batches**	**Mean±SEM (mV)**	**Statistics**	**Sign. (p value)**	**Sign**		

**WT**	24/7	168.93±1.47	one-way ANOVA				
**F489A**	9/3	140.25±1.93		<0.001 (vs. WT)	***		
**L493A**	10/3	138.16±1.48		<0.001 (vs. WT)	***		
**L530A**	9/3	142.92±1.46		<0.001 (vs. WT)	***		
**<u>Fig 8I</u>**	**Cells/Batches**	**Mean±SEM (mV)**	**Statistics**	**Sign. (p value)**	**Sign**		
**WT**	24/7	168.93±1.47	one-way ANOVA				
**R295A**	7/2	224.29±3.22		<0.001 (vs. WT)	***		
**Q452A**	8/3	210.76±5.14		<0.001 (vs. WT)	***		
**R501A**	8/3	204.64±3.64		<0.001 (vs. WT)	***		
**N505A**	8/3	192.73±4.21		<0.001 (vs. WT)	***		
**F529A**	9/3	205.18±2.52		<0.001 (vs. WT)	***		
**Q446A**	5/2	165.73±2.87					
**<u>Fig S5B</u>**	**Cells/Batches**	**Mean±SEM (pA/pF)**	**Statistics**	**Sign. (p value)**	**Sign**		
**WT 11 mM**	7/2	5.01±0.62	one-way ANOVA	0.022 (vs. 51 mM)	*		
**51 mM**	9/3	10.1±1.41		<0.001 (vs. 161 mM)	***		
**161 mM**	24/7	25.99±1.36		0.033 (vs. 221 mM)	*		
**221 mM**	8/2	37.86±2.80					
**<u>Fig S5E</u>**	**Cells/Batches**	**Mean±SEM (pA/pF)**	**Statistics**	**Sign. (p value)**	**Sign**		
**pH$_O$8.8**	13/3	9.63±1.02	one-way ANOVA				
**pH$_O$7.5**	24/7	25.99±1.37		<0.001 (vs. pH$_O$8.8)	***		
**pH$_O$6.5**	15/4	42.08±2.34		0.008 (vs. pH$_O$7.5)	**		
**pH$_O$5.5**	20/5	50.99±3.18		0.06 (vs. pH$_O$6.5)			

**Fig S5F**	**Cells/Batches**	**Mean±SEM (pA/pF)**	**Statistics**	**Sign. (p value)**	**Sign**		
**pH_O8.8**	13/3	-1.3±0.28	one-way ANOVA				
**pH_O7.5**	24/7	-4.67±0.51		0.08 (vs. pH_O8.8)			
**pH_O6.5**	15/4	-9.56±0.81		<0.001 (vs. pH_O7.5)	***		
**pH_O5.5**	20/5	-23.43±1.65		<0.001 (vs. pH_O6.5)	***		
**Fig S5H**	**Cells/Batches**	**Mean±SEM (ms)**	**Statistics**	**Sign. (p value)**	**Sign**		
**pH_O7.5**	24/7	280.09±9.93	one-way ANOVA				
**pH_O6.5**	15/4	215.87±14.47		<0.001 (vs. pH_O7.5)	***		
**pH_O5.5**	20/5	161.09±8.11		0.006 (vs. pH_O6.5)	**		
**Fig S5J**	**Cells/Batches**	**Mean±SEM (ms)**	**Statistics**	**Sign. (p value)**	**Sign**		
**pH_O7.5**	24/7	25.36±1.45	one-way ANOVA				
**pH_O6.5**	15/4	43.72±3.14		<0.001 (vs. pH_O7.5)	***		
**pH_O5.5**	20/5	77.26±2.39		<0.001 (vs. pH_O6.5)	***		
**Fig S9B**	**Cells/Batches**	**Mean±SEM (pA/pF)**	**Statistics**	**Sign. (p value)**	**Sign**		
**WT**	24/7	25.99±1.36	unpaired Student's t-tests				
**Y553C**	13/3	39.80±2.17		<0.001 (vs. WT)	***		
**Fig S9C**	**Cells/Batches**	**Mean±SEM (mV)**	**Statistics**	**Sign. (p value)**	**Sign**		
**WT**	24/7	168.93±1.47	unpaired Student's t-tests				
**Y553C**	13/3	123.9±3.14		<0.001 (vs. WT)	***		
**Fig S10C**	**Cells/Batches**	**Mean±SEM (mV)**	**Statistics**	**Sign. (p value)**	**Sign**		

**WT**	24/7	168.93±1.47	one-way ANOVA				
**F317Y**	8/2	173.82±1.45					
**F317W**	8/2	172.05±1.84					
**F317A**	12/3	133.21±2.56		<0.001 (vs. WT)	***		
**Fig S10D**	**Cells/Batches**	**Mean±SEM (ms)**	**Statistics**	**Sign. (p value)**	**Sign**		
**WT**	24/7	280.09±9.93	one-way ANOVA				
**F317Y**	8/2	93.11±10.73		<0.001 (vs. WT)	***		
**F317W**	8/2	76.01±8.55		<0.001 (vs. WT)	***		
